# Metabolic Reprogramming and Reconstruction: Integration of Experimental and Computational Studies to Set the Path Forward in ADPKD

**DOI:** 10.3389/fmed.2021.740087

**Published:** 2021-11-24

**Authors:** Roberto Pagliarini, Christine Podrini

**Affiliations:** Molecular Basis of Cystic Kidney Disorders Unit, Division of Genetics and Cell Biology, IRCCS-San Raffaele Scientific Institute, Milan, Italy

**Keywords:** metabolism, systems biology, systems medicine, therapeutics, drug repositioning, ADPKD

## Abstract

Metabolic reprogramming is a key feature of Autosomal Dominant Polycystic Kidney Disease (ADPKD) characterized by changes in cellular pathways occurring in response to the pathological cell conditions. In ADPKD, a broad range of dysregulated pathways have been found. The studies supporting alterations in cell metabolism have shown that the metabolic preference for abnormal cystic growth is to utilize aerobic glycolysis, increasing glutamine uptake and reducing oxidative phosphorylation, consequently resulting in ADPKD cells shifting their energy to alternative energetic pathways. The mechanism behind the role of the polycystin proteins and how it leads to disease remains unclear, despite the identification of numerous signaling pathways. The integration of computational data analysis that accompanies experimental findings was pivotal in the identification of metabolic reprogramming in ADPKD. Here, we summarize the important results and argue that their exploitation may give further insights into the regulative mechanisms driving metabolic reprogramming in ADPKD. The aim of this review is to provide a comprehensive overview on metabolic focused studies and potential targets for treatment, and to propose that computational approaches could be instrumental in advancing this field of research.

## Introduction

Autosomal Dominant Polycystic Kidney Disease (ADPKD) is a common monogenic disorder characterized by bilateral renal cyst formation and extra-renal manifestations. These manifestations include cysts in other organs, such as the liver and pancreas, as well as abnormalities including intracranial aneurysms. Worldwide around 12.5 million people suffer from ADPKD, and it is a common cause of end-stage renal disease (1). ADPKD is caused by mutations in either *PKD1* (polycystin-1) or *PKD2* (polycystin-2) genes, which account for almost 80 and 15% of ADPKD cases, respectively. More recently, a third gene has been described, *GANAB* (which encodes neutral alpha-glucosidase AB), which results in a milder renal phenotype and a more severe liver phenotype (2).

The polycystin1 (PC1) protein contains a large extracellular N-terminal region (3074 aa), 11 transmembrane domains (1032 aa), and a short intracellular C-terminal region (198 aa) (3, 4). PC1 interacts with PC2 through a coiled-coil domain in the C-terminal portion (5). The PC1 and PC2 complexes are detected in the ER membrane, the plasma membrane, exosomes, and the primary cilia, where they have been suggested to mediate calcium influx in response to fluid flow (6). Notwithstanding, the function of polycystin proteins in cilia and their role in calcium fluxes has been intensely debated (7).

The polycystin proteins have been shown to modulate numerous signaling pathways [please refer to this review for evidence of altered signaling pathways (8)] however their function remains unclear. In the last decade, research aimed at elucidating metabolic-related pathways in ADPKD has grown exponentially, and metabolic reprogramming is now considered a main feature of ADPKD (9–12). The number of researchers using multiple-omics (metabolomics, transcriptomics, genomics, and lipidomics) approaches is still growing and these studies have been pivotal in gaining substantial insights into the pathogenesis of PKD. There is a need for the scientific community to establish principles for data management, which can be challenging to interpret, unless efficiently analyzed. This huge amount of data creates more confusion and blurring than understanding.

**Figure 1:**
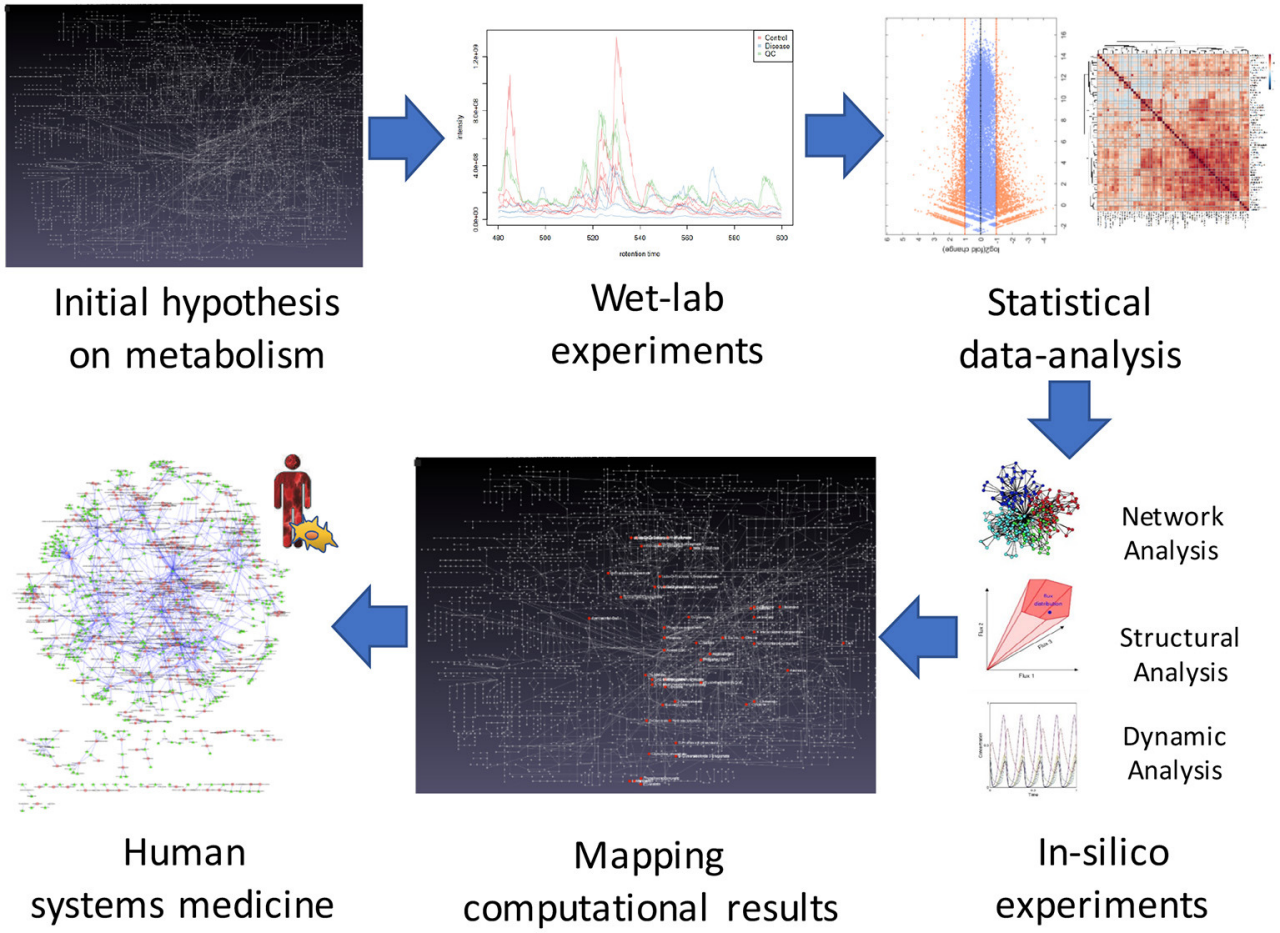
The systems biology approach in ADPKD. Steps of the workflow for studying possible new therapeutics targets for ADPKD. The first step concerns the formulation of an initial hypothesis on possible changes in a metabolic network due to the disorder. In the second step, wet-lab experiments are employed to obtain omics data. The third step involves statistical analysis of the experimental data that will be used in the fourth phase to develop mathematical models for *in-silico* simulations. In the fifth step, in order to validate the initial hypothesis, the simulation results are mapped to the metabolic network. Finally, systems medicine is employed to propose new possible therapeutic targets.

One solution to overcome this problem is through the integration of Systems Biology approaches, which offer powerful abstraction tools to analyze biological reactions in a cell, providing a snapshot that is similar to its observable phenotype. Computational tools can be used not only to explore comprehensive molecular mechanisms but also to discover and test-novel therapeutic targets. For example, they were recently applied to identify novel drug candidates for the treatment of ADPKD (13, 14). A possible System Biology workflow for the development of possible therapeutic targets that integrates wet-lab experiments, statistical and computational analysis, and System Medicine, is proposed in Figure 1. Systems Medicine represents a novel, more personalized, precise, and systematic, interdisciplinary approach in medicine. It involves the implementation of computational approach in medical concepts, research, and clinical practice, through iterative and feedback exchanges between clinicians, biologists, pharmacologists, bioinformaticians, and mathematicians.

## Metabolic Changes in ADPKD

A broad range of metabolic pathways in ADPKD were described including: increased glycolysis, reduced fatty acid oxidation (FAO) and altered glutamine utilization with defective mitochondrial function. We describe the current biological findings and how mathematical modeling applied to biological data can be useful to gain further insights into the metabolic alterations.

### Lactate as an Energy Source

One of the common features of cystic cells derived from murine models of ADPKD is that they exhibit increased glucose uptake and lactate accumulation (9). In *Pkd1*^−/−^ cells, glucose is converted to pyruvate which is then reduced to lactate instead of being transported into mitochondria (9, 12). Metabolic tracing with ^13^C_6_-glucose by NMR revealed a significant increase in lactate production in cystic kidneys compared to age-matched controls derived from an aggressive model (*Ksp-Cre:Pkd1*^*flox*/−^) and a slowly progressive murine model (*Tm-Cre:Pkd1*^*flox*/−^) of ADPKD (9). Increased glucose uptake was also found in *Pkd1* mutant mouse embryonic kidney (MEK) cells compared with *Pkd1* WT MEK cells (15). Further, an upregulation of glycolytic markers was observed (9, 15, 16), and the glycolytic signature was confirmed in a subset of human microarray data and cystic kidneys derived from hypomorphic *Pkd1* mutant mice (9, 12, 17). A recent study investigated the cross-sectional association of urinary metabolic biomarkers with ADPKD (18) severity among the TAME-PKD trial participants at baseline (19) (Please refer to Table 1 for mentioned clinical trials). This study is the first evidence of human urine samples supporting that increasing ADPKD severity is associated with a metabolic shift toward increased aerobic glycolysis. Other studies on the analysis of biomarkers in ADPKD should follow as to determine the ability to detect if a particular biomarker changes with time and if the correlations may be associated with cellular damage rather than metabolic changes *per se*. The enhanced glycolysis, the so-called Warburg effect reminiscent of cancer cells (49), contributes to the metabolic re-wiring in ADPKD and is considered a prominent feature of this disease.

**Table 1:** Characteristics of the included clinical trials.

**Drug**	**ClinicalTrials.gov Identifier**	**Publications indexed to study**	**Population**	**Outcome**
Metformin	NCT02656017	(19)	Adult 18 to 60 years	Completed
Pravastatin	NCT00456365	(20–25)	Child, Adult 8 to 22 years	Completed
Pravastatin	NCT03273413	(26)	Adult 25 to 60 years	Recruiting
Lisinopril, Telmisartan	NCT00283686	(27–30)	Child, Adult 15 to 64 years	Completed
Lisinopril, Telmisartan	NCT01885559	(31)	Child, Adult 15 to 64 years	Completed
Sirolimus	NCT01223755	(32)	Adult, Older Adult 18 to 80 years	Completed
Sirolimus	NCT00346918	(33–37)	Adult 18 to 40 years	Completed
Rapamune	NCT00286156		Adult, Older Adult 18 to 75 years	Completed
Everolimus	NCT00414440	(38)	Adult 18 to 50 years	Completed
Rapamycin (sirolimus)	NCT00920309		Adult, Older Adult 18 to 70 years	Terminated
Lisinopril, Telmisartan	NCT01885559	(31)	Child, Adult 15 to 64 years	Completed
Pravastatin	NCT00456365	(20–25)	Chid, Adult 8 to 22 years	Completed
Lisinopril, Telmisartan	NCT00283686	(27–30)	Child, Adult 15 to 64 Years	Completed
Lisinopril,Telmisartan	NCT01885559	(31)	Child, Adult 15 to 64 Years	Completed
RGLS4326	NCT04536688		Adult, Older Adult 18 to 70 years	Recruiting
Fasting/ketogenic^*^	NCT04472624	(39)	Adult 18 to 60 years	Active, not recruiting
Tolvaptan	NCT00428948	(40–48)	Adult 18 to 50 years	Completed

*Order indicated as in the review*.

* *intervention/treatment*.

### Mitochondrial Abnormalities and ER Cross-Talk

Mitochondria are the central organelles for metabolism and are the key players for energy production in the cells. Mitochondrial respiration has been reported to be altered in *Pkd1*^−/−^ proximal tubule cells (11). Indeed, the investigators demonstrated that oxygen levels regulate the localization and activity of PC1 by a prolyl hydroxylase domain-containing protein 3 (EGLN3). Furthermore, the study reported that the polycystin proteins localize in the mitochondria-associated membranes (MAMs), where they can mediate calcium signaling from the ER lumen to the mitochondrial matrix (11). PC2 depletion alters calcium transfer and decreases mitochondrial movement. This decrease is thought to be the result of an increased expression of the ER-mitochondrial tethering protein, mitofusin 2 (MFN2), which ultimate affecting calcium-mediated mitochondrial bioenergetics that contribute to cell proliferation (50). These findings collectively suggest that the polycystins are important regulators of mitochondrial function, but whether mitochondrial calcium and dynamics are disrupted in *PKD1* mutations warrants further investigation. A cleaved carboxyl-terminal fragment of PC1 has been proposed to accumulate in mitochondria, indicating that PC1 may directly modulate mitochondrial function (51). However, how this cleavage is involved in cyst formation or mitochondrial function is still unknown.

Morphologically, abnormal mitochondria were described in cyst-lining cells in an ADPKD mouse model (*Ksp-Cre:Pkd1*^*flox*/*flox*^) and in rats (Han:SPRD *Cy/*+) (52). This was accompanied by a reduction in mitochondrial number suggesting a decrease in mitochondrial mass in the cystic epithelia (52). In another study, the decrease in mitochondrial mass was found to be correlated with a reduced activity of mitochondrial respiratory enzymes and fragmentation of the mitochondrial network in the cystic epithelial of *Ksp-Cre:Pkd1*^*flox*/−^ kidneys (53). The inhibition of mitochondrial function was shown to be mediated by miR-17- *Ppar*α axis (54). The miR-17 inhibits OXPHOS and fatty acid oxidation, and its suppression improves mitochondrial function of cyst epithelia (54). PPARγ, another member of the PPAR subfamily has been shown to be implicated in ADPKD pathogenesis. Moreover, treatment with PPARγ agonist rescues proliferation and retards cyst growth in rodent PKD models (55–57).

The master regulator of mitochondrial biogenesis, peroxisome proliferator-activated receptor γ coactivator 1 α (PGC-1α), was downregulated in kidney tissues from *Pkd1*^*flox*/*flox*^ and 7-week-old *Cy/*+ rats (52). PPARA and its target genes were shown to belong to a down regulated pathway in human ADPKD cysts (17). Interestingly, the mitochondria from PC2 knock-down (KD) cyst-modeling cells exhibit altered metabolic capacity and show a high degree of fragmentation, which they have in common with *PKD1*; however, an increased level in PCG-1α was shown in PC2 KD cells (50). The reasons for these different phenotypes between cells lacking PC1 and PC2 proteins still remains unclear, but the idea that mitochondria-ER communication is a common mechanism that contributes to altered metabolism in ADPKD is well-evidenced.

**Figure 2:**
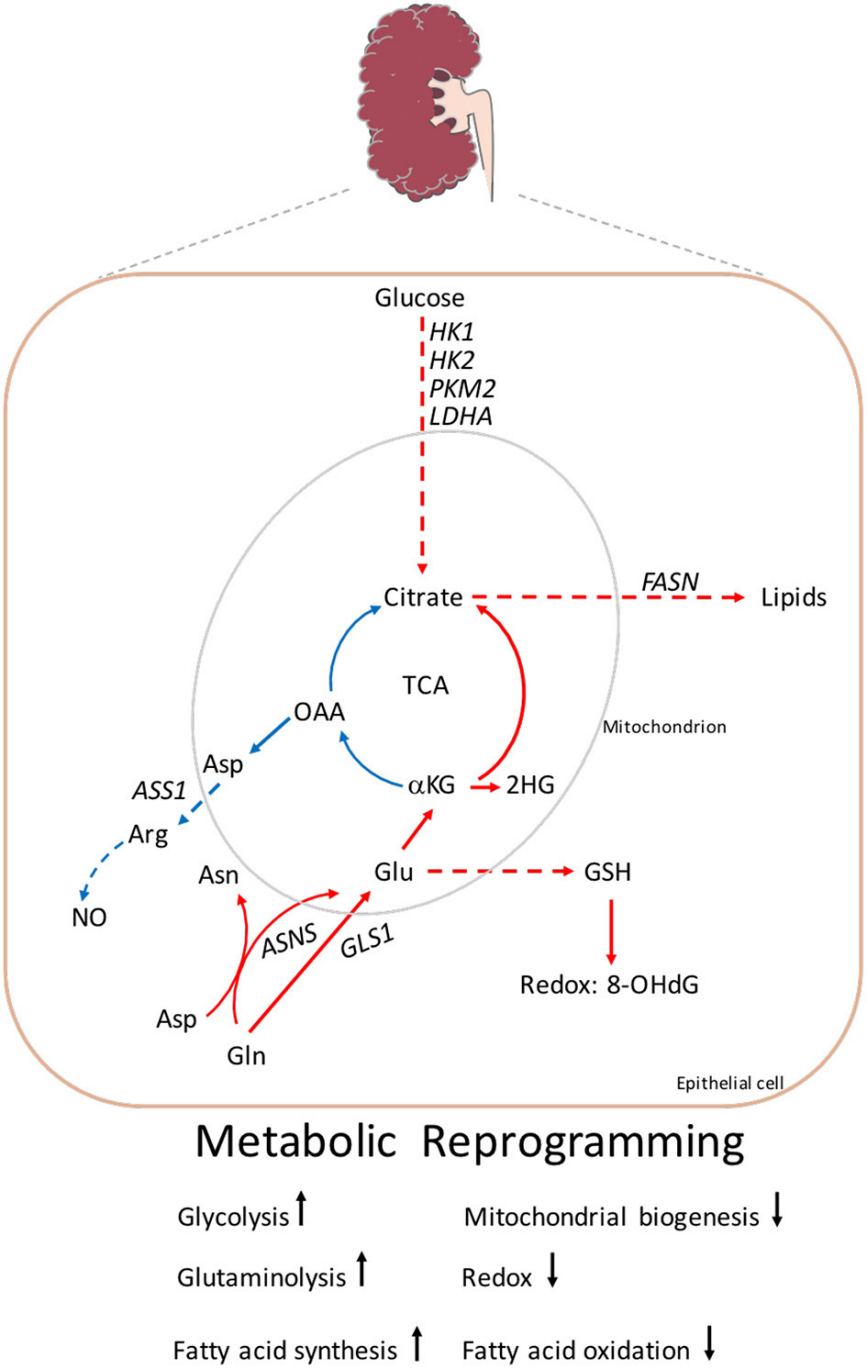
Metabolic reprogramming in ADPKD. A screen shot of the main metabolites and key genes altered in ADPKD described in the review. Enhanced glycolysis which leads to the production of lactate. Increased glycolytic genes: *HK1, HK2, PKM2*, and *LDHA*. The TCA mitochondrial metabolites OAA and citrate are used to generate aspartate and acetyl-CoA, the latter for the production of lipids. Aspartate together with citrulline (not shown) are converted to arginine by ASS1, which synthesizes NO. The uptake of glutamine is driven by GLS, and/or through ASNS. Glutamine contributes to the generation of GSH which attenuates 8-OHdG, a product of DNA damage. Glutamine is also necessary for the production of glutamine sourced 2-HG. Gln, glutamine; Glu, glutamate; αKG, α-ketoglutarate; 2-HG, 2-hydroxyglutarate; OAA, oxaloacetate; Asn, asparagine; Asp, aspartate; Arg, arginine; NO, nitric oxide; GSH, glutathione; 8-OHdG, 8-hydroxyguanosine; *FASN*, fatty acid synthetase; *GLS1*, glutaminase1; *ASS1*, arginosuccinate synthetase 1; *HK1*, hekoxinase1; *HK2*, hexokinase2; *PKM2*, pyruvate kinase M2; *LDHA*, lactate dehydrogenase. Arrow indicates metabolic reactions, while dashed lines represent pathways linking two metabolites. Color code: upregulation red, blue downregulation.

The major pathway for the degradation of fatty acids inside the mitochondria is mitochondrial fatty acid β-oxidation (FAO), which is the preferred energy source for highly metabolic cells and can contribute to mitochondrial dysfunction. Under physiological conditions, FAO provides more ATP than glucose oxidation (58). In *Pkd1* knock-down cells derived from mouse cortical collecting duct (mCCD) and from immortalized proximal or distal tubule cells from *Pkd1*^*cko*/*cko*^, the oxygen consumption rate (OCR) was reduced in the presence of palmitate compared to respective controls (10). The reduced FAO, observed by OCR, was shown in other cell lines derived from ADPKD mouse models (11, 12, 54). Often, this reduction is accompanied by increased fatty acid biosynthesis, which can favor the increased *de novo* fatty acid synthesis (FASN) and inhibit the increased synthesis of carnitine palmitoyl transferase 1 (CPT1) transporters into the mitochondria. This was observed in *Pkd1*^−/−^ cells and kidneys from *Ksp-Cre:Pkd1*^*flox*/−^ when compared to controls (12). These results suggest that fatty acids derived carbons sustain reductive carboxylation for the *de* n*ovo* fatty acids biosynthesis through glutamine entry into the tricarboxylic acid cycle (TCA), and may also sustain the energy required for cell membranes used for proliferation. The increased in fatty acid biosynthesis was also sustained from an upregulation of key genes involved in this process derived from a subset of microarray data from human ADPKD patients (12). However, the precise mechanism of how PC1 regulates the mitochondrial metabolism is still not precisely defined and understanding the origin of these perturbations including the role of the polycystin in the regulation of the mitochondrial function needs to be defined (please refer to Figure 2 for summary of the main metabolic pathways altered in ADPKD).

### Glutaminolysis and Amino Acid Metabolism

Glutamine is another important metabolite that sustains the supply of carbons and nitrogens to fuel biosynthesis of the TCA cycle metabolites (59). In standard culture, many cancer cell lines fuel the TCA cycle using acetyl-CoA produced from glucose and most of the anaeplerosis is supplied by glutamine (60). Glutamine addiction in ADPKD was first reported in the study by Hwang et al. In this study, a non-targeted metabolomics approach was investigated in autosomal recessive polycystic kidney disease (ARPKD) kidneys, and *cpk* (congenital polycystic kidneys) cells. The results were then validated *via* targeted analysis with changes in human ARPKD tissue and unaffected controls (61). *Cpk* cells are addicted to glutamine, which is required for the production of the oncometabolite glutamine-sourced 2-hydroxyglutarate (2-HG) (61). Accumulation of 2-HG has been described in renal cell carcinoma and it is thought to contribute to enhanced proliferation through epigenetic modifications (62). This striking parallel between cancer and ADPKD was firstly evidenced in this study (61) and subsequently associated with profound metabolic reprogramming that is found in tumors. However, ADPKD does not predispose to malignancy and the reader can find further details in the following review (63).

The glutamine addiction in ADPKD opened novel therapeutic opportunities, and subsequent studies have shown that inhibition of glutaminase 1 (GLS1) *via* BPTES or CB-839, tested in a less aggressive model of cystic disease (*Aqp2-Cre:Pkd1*^*fl*/*fl*^ and *Pkhd1-Cre: Pkd1*^*fl*/*fl*^) (64), effected proliferation and cyst growth. The inhibition of glutamine with CB-839 resulted in cystic amelioration in *Aqp2-Cre: Pkd1*^*fl*/*fl*^ but not in *Pkhd1-Cre: Pkd1*^*fl*/*fl*^ mice. The investigators identified differences in signaling pathways due to mTORC1 and ERK activation, which might explain the likely failed inhibition, suggesting the upregulation of an adaptive metabolic pathway is able to bypass GLS1 inhibition. In another study by Podrini et al., tracing studies with [^13^${\text{C}}_{5}^{15}$N_2_] glutamine revealed that *Pkd1*^−/−^ cells uptake more glutamine by utilizing the enzyme asparagine synthetase (ASNS) (12) (please refer to Figure 2). ASNS is a transamidase that converts aspartate into asparagine while deaminating glutamine to form glutamate (65). ASNS was found to be upregulated in cells lacking the *Pkd1* gene, in cystic kidneys derived from *Ksp-Cre:Pkd1*^*flox*/−^, and in a subset of microarrays from humans and murine kidneys when compared to the corresponding controls (12). To evaluate the relevance of *ASNS* upregulation in PKD, silencing *Asns* in *Pkd1* devoid cells completely rescued the accumulation of α-ketoglutarate (α-KG), specifically reducing the contribution to glutamine-derived α-KG (12). One possible mechanism for the increased in ASNS is that asparagine synthesis supplies the availability of the TCA cycle intermediates and supplies the reduced nitrogen to maintain the pool of non-essential amino acid synthesis (66) but this needs to be investigated in ADPKD. In a metabolic profiling performed in children and young adults with ADPKD, Plasma samples were collected analysed with mass spectrometry (MS) and the most influential metabolite in ranked based area was asparagine, which was more than threefold higher in patients with ADPKD (67). In another study, glutamine addiction in PKD was investigated by an alternative glutamine pathway, *via* arginine synthetic enzyme arginosuccinate synthase1 (ASS1) directed to urea production (68). In both mouse models and human ADPKD tissue, the authors demonstrated that ASS1 expression was reduced and arginine depletion resulted in decreased cyst formation (68). Targeting arginine, *via* arginine-degrading enzymes, as used in clinical trials, and reviewed in Patil et al. (69), could be used to reduce circulating arginine in PKD. Collectively, these results suggest that ADPKD depends on glutamine for growth, shows increased asparagine synthetase activity, and its inhibition reduces cells growth (12).

Glutamine is also a precursor for the major antioxidant system compromised of glutathione (GSH) and oxidized glutathione (GSSG) (70). In another study by Flowers et al., the investigators found that embryonic *Pkd1* mCCD are also dependent on glutamine, which is required for glutathione synthesis (71). The changes in glutathione metabolism and the vulnerability of mutant *PKD* cells to reactive oxygen species (ROS) was found at a very early stage of PKD, which was reflected by increased 8-hydroxydeoxyguanosine (8-OHdG) that enhanced disease progression (72). The oxidative stress biomarker 8-OHdG was increased in the kidney cyst-lining cells from *Ksp-Cre:PKD1*^*flox*/*flox*^ mice and *Cy/*+ rats, indicating that mitochondrial abnormalities also incite oxidative stress in these cells (52). There has been a link between oxidative stress and impaired endothelial-dependent relaxation (endothelial dysfunction) in progression of kidney injury with ADPKD, as observed in Nowak et al. (73) and Klawitter et al. (74). The PC1/PC2 complex is necessary for normal vasculature development, since it is required for endothelial cilia to sense fluid shear stress through complex biochemical cascades involving many factors including nitric oxide (NO) (74). NO is produced by endothelial cells and is a critical regulator of this balance, such that endothelial dysfunction is defined as reduced capacity for NO production (75). Endothelial dysfunction and decreased endothelial NO synthetase activity are observed in patients (74) and young adults with ADPKD (73). Because statins have anti-inflammatory and anti-oxidant effects (76), there have been studies for the prevention of acute kidney injury after cardiac surgery (77) which predisposes to endothelial dysfunction. In a pediatric ADPKD trial, plasma levels of oxidative stress biomarkers declined during pravastatin treatment but not with placebo (78). There is also an ongoing clinical trial in adults (Pravastatin in adult phase 4, ClinicalTrials.gov Identifier: NCT03273413). However, a *post hoc* analysis was performed on adults in the HALT-PKD trials on statins resulting in no benefit from statin therapy (79). Whether oxidative stress is a driver of disease still needs further investigation.

### Mathematical Modeling of Metabolism: Application to ADPKD

At the genome-scale level, metabolism is the result of thousands of compounds that are linked by reactions, which are continuously interacting with each other. The mathematical modeling process of metabolic networks can be broken in two steps. First, the stoichiometry of the system, which is the set of reactions representing the species connectivity, is established, and then rate laws are assigned to each reaction. For specific pathways, kinetic models based on sets of ordinary differential equations (ODEs) are broadly used to describe the metabolites' dynamic along time (80, 81). The foundation of this approach is the theory of molecular collisions that represents the basis of the mass action law. According to this theory, the algebraic expression for the ODEs depends on the reaction kinetics under consideration. There are several approaches for deriving kinetic functions: mass action kinetics (82), the Michaelis-Menten type and allosteric kinetics (83, 84), and the power-law approximation (85). The major drawback in using ODEs is the scarcity of experimental data on rate constants, especially at the genome-scale level. Genome-scale metabolic models require a large number of kinetic constants, many of which have not yet been experimentally measured. Moreover, the value of these parameters are subject to high uncertainty, resulting in erroneous simulations. Stoichiometric models address these problems by assuming that the changes in the compound concentrations are the result of the topological structure of the metabolic reactions. Starting from this assumption, by applying Flux Balance Analysis (FBA), a set of ODEs is transformed into a system of linear equations, and its rates can be obtained by solving a linear programming problem that optimizes specific objective functions (86). Further constraints are added to narrow the solution space. Although these types of models do not depend on the mathematical formalization of the rate laws, their analysis can lead to general results about the reactions properties. In one study (12), a computational systems-level approach based on FBA, called Differential Flux-balance Analysis (DFA) (87, 88), was employed to gather a broader understanding of the metabolic changes observed in *Pkd1* mutant cells. By employing tracing experiments with ^13^C-labeled glucose, the investigators found an 1.6 fold increase in the uptake of glucose in *Pkd1*^−/−^ cells compared to controls. This data was used as mathematical constraint for DFA. The simulations suggested that an increased uptake of glucose is able to drive a statistically significant change of the bioenergetics mechanisms, such as the TCA cycle, oxidative phosphorylation, the pentose phosphate pathway, and fatty acid synthesis and oxidation. The simulations have been followed by an extensive study of the observed metabolic reprogramming by integrating *in vitro* and *in vivo* analysis with metabolomics and transcriptomics data. In a subsequent work (89), DFA has been applied to test whether these alterations are causally interconnected or if they might occur simultaneously without interfering with each other. Particularly, DFA has been integrated with a Compounds-Pathways Enrichment Analysis to simulate the metabolic changes of bioenergetics pathways observed in Podrini et al. (12): (i) increased glucose uptake; (ii) increased mitochondrial glutamine uptake; (iii) increased fatty acids synthesis; (iv) reduced fatty acids oxidation. They found that simulations of these alterations result in important overall metabolic changes in pathways that are very similar in the different conditions, suggesting that they are all interconnected by key genes and specific chemical reactions. However, slightly different alterations were observed in the different simulations and only increased glycolysis appears to be the alteration that better recapitulates simultaneously all changes observed in *Pkd1* mutant cells, while none of the other simulations can recapitulate the overall picture, suggesting an important possible causal link and directionality of the alterations observed. In addition, DFA showed that an increased flux of citrate from mitochondria to cytosol modulates the switch toward alternative bioenergetics pathways, hence, this metabolite could be one of the key factors in the regulation of the metabolic alterations observed in ADPKD.

Computational analysis may contribute to basic research on ADPKD, but the success of this approach will certainly require new modeling and simulation tools because our current understanding of how regulations are carried out is probably still missing several significant pieces. More experimental work is needed, and these experimental results must be incorporated in improved mathematical models, which would benefit from the creation of a common modeling framework that takes into account different entities, such as genes, proteins, and metabolites, and relationships, like metabolic reactions, interactions, regulations, and transports. Several problems and requirements arise toward this strategy, such as how to deal with incomplete information, how to manipulate large models, how to extract valuable information about the regulative mechanisms, how to analyze these models, and how to infer suitable models from experimental data.

## Systems Biology of ADPKD

The goal of Systems Biology is to try to understand global dynamics and properties of biological processes on the basis of microscopic factors depending on the scale of the phenomenon under study. The unifying framework is that biological systems are the result of a high number of interacting elements that are organized, at different levels of complexity, in functional networks. Particularly, the availability of high-throughput data about multiple omics allows mapping cellular ADPKD processes at the level of genes, proteins, and metabolites.

The work of Song et al. (17) represents the first application of a Systems Biology approach in ADPKD. By employing microarray expression profiling from cysts of different size and from minimally cystic tissue from five *PKD1* human polycystic kidneys, the authors identified that a cross-talk of specific signaling pathways modulates renal cyst expansion. They inferred downregulation of several gene sets associated with metabolic pathways like amino acids, fatty acids, urea cycle and ATP metabolism, and other kidney specific gene sets associated with ciliary functions, and renal cystic diseases. In contrast, sets of signaling genes resulted upregulated. The database developed by Song et al. still represents an invaluable resource for researchers interested in studying molecular pathobiology of ADPKD, and it was employed in several computational approaches related to ADPKD. This dataset has been analyzed in Rahimmanesh and Fatehi (90) by employing differentially expressed genes (DEGs) to construct protein-protein interactions (PPIs) networks. Enrichment analysis applied to protein clusters found in networks resulted in the detection of critical metabolic pathways such as oxidative phosphorylation and energy metabolism. The inferred networks also demonstrated that the interactions among proteins become more complex with the disease progression, while clustering revealed that EDN1, a vasoconstrictor that may promote tumor growth and for which serum levels have been associated with disease progression in ADPKD (91, 92), seems to be a seed gene in the early stage of the disorder. In addition, miRNAs and transcription factors enriched for dysregulated genes have been determined, such as hepatocyte nuclear factor alpha (HNF4A). In another study (93), the Song's database has been integrated with a mice microarrays datasets (17, 94), and a set of DEGs was found in both the datasets. The downregulated genes were associated with carbon metabolism, fatty acids, and amino acid metabolism. Pathways involved in cancer, PI3K-Akt signaling pathway, focal adhesion, complement, and coagulation cascades resulted enriched for the upregulated DEGs. Starting from the DEGs, the authors filtered out a PPI network containing two significant modules. One module contained proteins related to mitochondrion, degradation of valine, lysine, leucine, fatty acids, ATP binding, and metabolism of carbon, propanoate, and tryptophan. The second one was associated with protein binding, extracellular region, extracellular space, platelet degranulation, extracellular exosome, plasma membrane, and aging (93).

With the aim of revealing the gene expression signatures related to progression of ADPKD, to infer signaling pathways associated with dysregulation of gene expression, and to study the roles of miRNAs in regulating the differences between normal and cystic kidneys, Pandey et al. investigated global microarray expression profiling (95). Kidneys from *Pkd1*^−/−^ littermates at the embryonic ages 14 (E14.5) and 17 (E17.5) were investigated and even if mutants at E14.5 do not exhibit cysts, specific changes in gene expression related to signaling were observed. This result indicates that gene expression changes at E14.5 could provide an indication of signaling pathways that cause cysts rather than being a consequence of cyst formation. Most of the deregulated gene sets in *Pkd1*^−/−^ mutants compared to WT at E14.5 and E17.5 were metabolic pathways and others involved in kidney development and regeneration. However, mutants at E17.5 days showed a broad change of genes associated with development, differentiation of specific nephron segments, and cyst formation and growth. Particularly, about 50% of the dysregulated pathways were in common with the deregulated ones observed in human ADPKD by Song et al. (17). In order to understand if miRNAs are directly involved in cyst formation and growth, miRNA microarrays using RNA from the kidneys of control and *Ksp-Cre:Kif3a* ^*flox*/*flox*^ mice was performed (96). The main goal of this work was the identification of differentially expressed miRNAs. Patel and colleagues showed that the oncogenic miRNA cluster miR-17~92 is upregulated in a mouse model of PKD. This miRNA drives cyst epithelial proliferation and inhibits the post-transcriptional expression of *Pkd1, Pkd2*, and hepatocyte nuclear factor 1β (*Hnf-1*β). An integrated analysis of miRNA and RNA-sequencing was employed in (94) to investigate the miRNA profiles correlated with the severity of cyst development in two ADPKD models, kidney-specific *Pkd1* or *Pkd2* KO mice, at three time points, P1, P3, and P7. Woo et al. discovered differentially expressed miRNAs that were common in both mouse models. Particularly, a small subset of these miRNAs have been associated with cytogenesis-related pathways. The role of miRNAs in ADPKD has also been recently studied in (97) by employing a transcriptional profiling of orthologous mouse models and cystic kidneys from humans with ADPKD. Lakhia et al. (97) showed that miRNAs represent molecular signals in the crosstalk between cyst microenvironment and immune cells. Particularly, miR-214 is upregulated in both mice and human PKD, while miR-214 deletion worsened cyst enlargement in the *Pkd2* KO murine model of ADPKD and increased cyst-related inflammation. This result suggests that miR-214 is a protective signal against cyst development and formation.

Since it has been observed that the kinetics of cyst formation after *Pkd1* inactivation in mice is sensitive to the age, specifically between days 12 and 14 (98), a network analysis has been applied to transcriptomics data from 36 mutants and 34 controls kidneys in order to study the changes during the P12-P14 interval that have such a drastic modifier effect in ADPKD (99). They inferred a cluster of DEGs enriched for several gene ontology categories related to metabolic pathways, development, cell differentiation, and anatomical structure morphogenesis. A gene ontology analysis suggested that metabolic pathways represent a key element in post-natal kidney development and the first step of cyst formation. The alteration of metabolic pathways has been confirmed by a metabolomics analysis of urine from 84 mice, which also showed an impairment of tyrosine metabolism, oxidative phosphorylation, purine metabolism, and aminoacyl-tRNA biosynthesis.

Finally, we would like to point out that long non-coding RNAs (lncRNAs) have emerged as epigenetic regulators of disorders and development. However, their role in ADPKD is not very clear yet. Therefore, RNA-sequencing analysis has been performed (100) with the aim to identify lncRNAs dysregulated in ADPKD. Several lncRNAs were deregulated in two kidneys specific *Pkd1* and *Pkd2* mutant mice. One of these, *Hoxb3os*, regulates a network of genes and signaling pathways that are commonly dysregulated in ADPKD. The ortholog *HOXB3-AS1* has been found to be downregulated in cystic kidneys from ADPKD patients. Several genes were differentially expressed between *Hoxb3os* KO and control cells. Pathway analysis suggested that *Hoxb3os* regulates mTOR pathway and metabolism in mouse kidney ADPKD cells. Specifically, *Hoxb3os* could represent a negative regulator of mTORC1 activity and mitochondrial respiration, suggesting that re-expression of *Hoxb3os* could be used as a therapeutic strategy in ADPKD.

In conclusion, it is important to highlight that the Systems Biology tools developed in the last few years represent powerful methods to generate systematic views of the ADPKD, with the aim of exploring the comprehensive molecular mechanisms of this disease.

### Emerging Therapeutics and Systems Medicine in ADPKD

The pathophysiology behind ADPKD is not completely understood, but a “threshold model” of cytogenesis is supported by previous studies (101–103). In this model, reduction of cellular levels of PC1 can influence cystic disease severity by a complexity of signaling pathways (104). However, re-establishing the levels of PC1 to restore normal cystic tissue remains an elusive approach. Targeting altered signaling pathways that drive cytogenesis (104) may be the preferred strategic approach to ameliorate disease progression in ADPKD. As a consequence of vasopressin V2-receptor activation, dyregulated signaling pathways in ADPKD such as increased cyclic adenosine monophosphate (cAMP) concentration, resulted in the identification of tolvaptan as a first treatment for ADPKD (105, 106). The activity of key energy metabolic enzymes was shown to be central in the metabolic shift of ADPKD. For example, AMP-activated protein kinase (AMPK) decreased (9, 107), while mTORC1 and ERK increased (108, 109). It was proposed that the decreased phosphorylation of AMPK is due to regulation of the liver kinase B1 (LKB1)/AMPK axis (9, 16). In another study, it was reported that the activation of the mitogen-activated protein kinase (MAPK)-ERK pathway was observed in an acute perinatal *PKD* model; however, an inhibitor against ERK was not able to prevent cyst formation and may not be the basis for cyst expansion (110). Inhibitors of the mTORC1 complex, including sirolimus and everolimus, have been tested in five clinical trials. The results of the clinical trials showed no beneficial effects on the rate of change of total kidney volume (TKV) (111).

A novel therapeutic approach that targets mRNA expression is to utilize the action of an antisense oligonucleotide (ASO), a single-stranded deoxyribonucleotide that is complimentary to the mRNA target. The goal of the ASO is the down regulation of a specific molecular target (112–114). Therapeutic use of ASOs has been approved by the FDA for various pathologies (115). An ASO target strategy has been tested by chronic administration of mTORC1 ASO in *Pkd2*^*W*25/−^ mice, a homologous model of human PKD caused by a mutation in the *Pkd2* gene, and resulted in improvement of the renal cystic phenotype (116). ADPKD also effects endothelial dysfunction (117) and circulating bioactive lipid mediators (118) which can contribute to metabolic disorders. From a clinical point of view, there is a higher incidence of new onset of diabetes mellitus after transplantation in recipients with ADPKD vs. other primary kidney diseases (119). To tackle a broad range of metabolic disorders in relation to vascular damage in ADPKD, clinical trials have been conducted with angiotensin-converting enzyme (ACE) inhibitors in ADPKD patients (31, 67). These trials have not been successful in decreasing renal function (120). Alternatively, the use of ASO against angiotensin (AGT) has been investigated in pre-clinical studies (116, 121, 122). The ASOs resulted in an improved cystic phenotype in the *Pkd2*^*WS*25^ model (123) and in the global (121) and in the tamoxifen-inducible knockout model of PKD (122). This suggests that targeting AGT with ASO may be more efficient than ACE inhibitors, and could open novel therapeutic opportunities for targeting abnormal signaling cyst formation in ADPKD.

**Figure 3:**
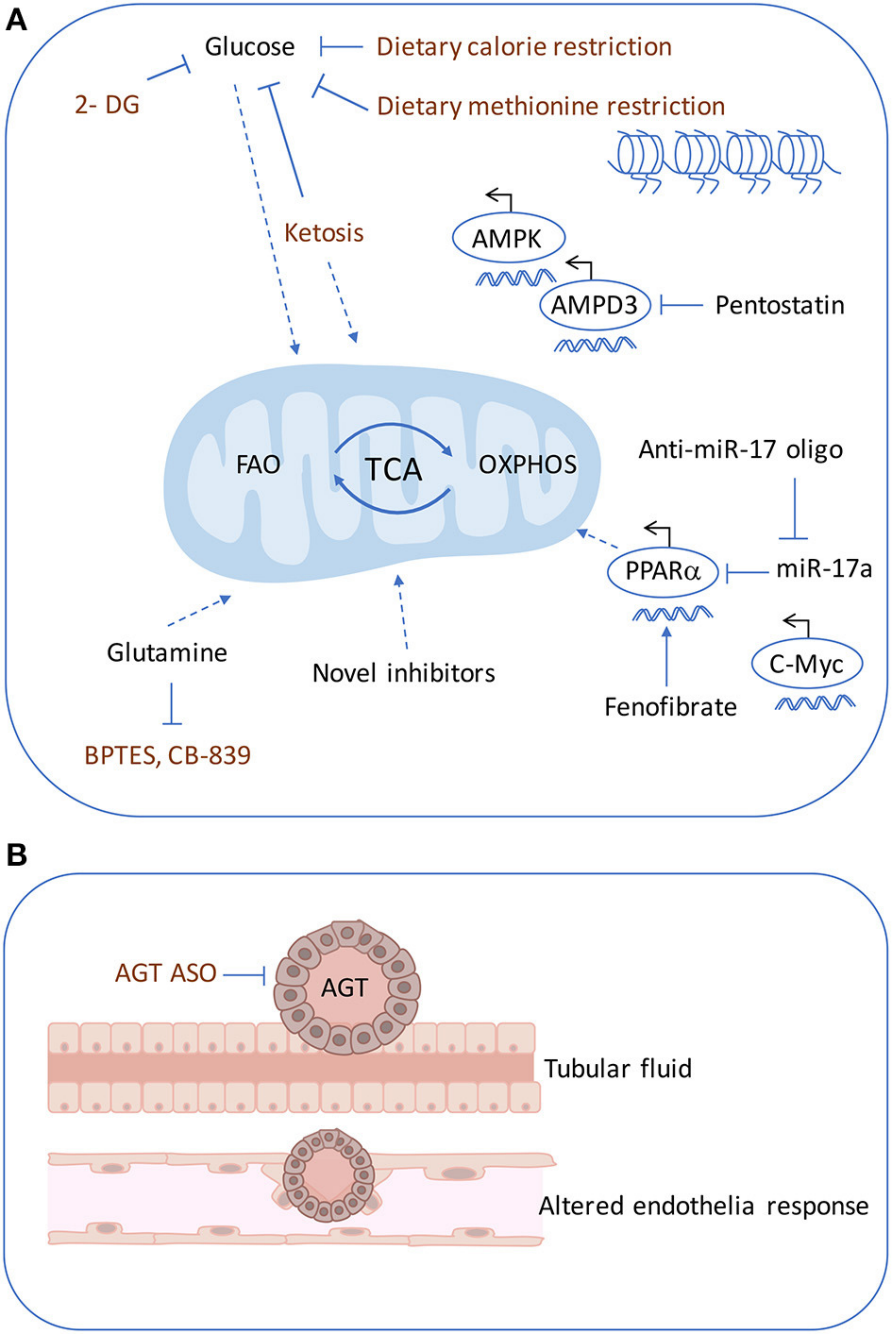
Therapeutics targets in ADPKD. Multiple pharmacological approaches are known to ameliorate cyst formation in ADPKD. **(A)** Glycolysis can be targeting using 2-DG and similarly with dietary restrictions such as, ketogenic diet, methionine restriction. The dietary restrictions also affect mitochondrial metabolism. OXPHOS defects can be rescued by using an anti-miR-17 oligonucleotide, PPARα inhibitor or fenofibrate PPARγ agonist. Super-enhancers driven cyst formation through AMPD3 can be inhibited by pentostatin. **(B)** Cyst expansion may compress the arterioles and trigger increased AGT causing altered endothelial response. ASO against, AGT, was shown to inhibit cyst formation. 2-DG, 2-deoxyglucose; AGT, angiotensin; ASO, antisense oligonucleotide.

The inhibition of mitochondrial function by anti-miR~17-92 attenuates disease progression in ADPKD mouse models irrespective of the mutated gene (*Pkd1* or *Pkd2*), the type of mutation (null or hypomorphic) or the dynamics of cyst growth (54). An anti-miR-17 oligonucleotide has proved to be efficient in reducing cyst formation in PKD mouse models (54, 124). Based on these results, anti-miR-17 oligonucleotides were designed and screened with favorable pharmaceutical properties (125). In fact, a phase 1 trial with an anti-miR-17 drug (RGLS4326) is currently being performed in ADPKD patients (ClinicalTrials.gov Identifier: NCT04536688). The miR-17 family is a promising drug target for ADPKD. Recent evidence shows that large clusters of transcriptional enhancers, super-enhancers (SE), undergo extensive remodeling during cytogenesis and that SE-associated transcripts are most enriched for metabolic genes in cystic cells (126). AMP deaminase 3 (AMPD3), a key enzyme involved in AMP metabolism and AMPK activation, was found to be SE-driven in cyst development. Inhibiting AMPD3 by pentostatin, or cyclic dependent kinase 7 (CDK7), one of the controlled metabolic target genes, decreased cell proliferation and apoptosis in ADPKD cells and delayed disease progression in tamoxifen-inducible *Pkd1* mice with slowly progressing ADPKD (126). These findings suggest that CDK7- containing SEs are critical in ADPKD. As for therapy, inhibiting AMPD3, could be beneficial to target before disease progression and can provide a greater efficacy than targeting the downstream effectors in ADPKD (please refer to Figure 3 for an overview of therapeutical targets).

Recent preclinical data have generated promising results for targeting cellular bio-energetics in ADPKD. The high rate of glycolysis encountered in ADPKD has been targeted with glucose analog 2-DG in orthologous mouse models of ADPKD (9, 107, 127) with effective cystic amelioration. A phase 1 clinical trial has been designed with 2-DG in an ADPKD cohort (128). In addition, the dependence on glucose in cystic cells opened new venues to target disease by modifying diet for cystic amelioration. In fact, 2-DG can be compared to calorie restriction without reducing food intake given its capability to reduce metabolic rate addiction to glucose (129). Thus, ketogenic diets that decrease glucose availability have been tested in ADPKD, with the hypothesis that ketogenesis may be beneficial in preventing cyst formation. This hypothesis was evaluated in an ADPKD rat model and in a spontaneous feline model of ADPKD, where a high ketogenic diet resulted in strong inhibition of PKD progression (39). The increase in ketosis was also investigated by acute fasting in mouse, rat, and feline models of PKD and significantly reduced renal cystic burden in all three models (39). Currently, there is an on-going clinical trial on the short term induction of ketosis in ADPKD patients (ClinicalTrials.gov Identifier: NCT04472624) and also the patent-pending supplements are also being formulated and will be tested in the near future in clinical trials (130). Furthermore, prior to testing the ketogenic diet calorie restriction was found to reduce cyst growth (131, 132). There is growing evidence that diet manipulation can be a modifier of ADPKD disease and that the plethora of metabolic dysregulation observed in ADPKD may support the proliferating cyst epithelia. One possible mechanism is through the impact of one-carbon and methionine metabolism which generate the essential methyl groups for which altered nutrient utilization is facilitated. Ramalingam et al. found a higher methionine-S-adenosyl methionine (SAM) levels and methyltransferase-like 3 (Mettl3) upregulation in multiple ADPKD mouse models and that methionine-SAM supplementation or *Mettl3* overexpression induced cyst growth (133). Dietary methionine restriction or Mettl3 ablation attenuated PKD, which may be regulated through epitranscriptomic mechanism through c-Myc and cAMP signaling activation (133). As for therapy, a vegan-based diet could also be used to ameliorate cyst formation, as methionine is found in meat and fish; alternatively, METTL3 inhibitors may also be targetable for ADPKD but warrants further investigation.

Tolvaptan, as already mentioned, is the unique drug that has been approved, in several countries, for the treatment of ADPKD (134). Frequent liver chemistry monitoring is suggested for patients using Tolvaptan, as it caused liver enzyme elevations in two pivotal Phase III clinical trials in ADPKD patients (42, 106). Taking into account this information, there is an urgent need for new compounds that can be used to stop the disorder progression.

A novel approach for identifying new drugs for ADPKD has been investigated by Malas et al. (14). Their approach combines integrated computational tools, repurposing analysis, cheminformatics data, and RNA sequencing of *Pkd1* KO mice at different disease stages in order to identify new drugs for ADPKD. Results from this approach indicated that three compounds (i.e., Meclofenamic acid, Gamolenic acid, and Birinapant) are able to inhibit cyst swelling *in vitro* without toxicity, and could be further investigated for the treatment of ADPKD. Meclofenamic acid targets the aldo-keto reductase family 1, which is involved in steroid metabolism (135), a pathway known to be a player in cyst development in a PKD rat model (136). Gamolenic acid has been selected based on PPARδ, a nuclear hormone receptor that controls a set of metabolic genes belonging to glucose metabolism, and fatty acid synthesis, storage, mobilization, and catabolism. Members of the PPAR family have been studied in pre-clinical trials for PKD (55, 137). Birinapant represents a second mitochondrial-derived activator of caspases (SMAC) mimetic that modulates apoptosis (138). It has been found that the SMAC mimetic GT13072 reduces PKD progression in a *Pkd1* mouse model (139).

Lixivaptan, another vasopressin V_2_ receptor antagonist, was proposed for ADPKD treatment. This compound was initially developed for treating hyponetremia (140), and it has been evaluated by applying quantitative systems toxicology modeling (141). Their results showed that when compared to Tolvaptan, Lixivaptan had a significantly lower risk of hepatotoxicity at their respective doses for ADPKD treatment; therefore, it is a good candidate for continued study in the treatment of this disorder. The difference in the toxicity mechanisms between Tolvaptan and Lixivaptan demonstrates the fact that even compounds within the same class can vary widely in terms of potential liability and potential mechanisms of toxicity.

Based on drug repositioning, Asawa et al. developed a high-throughput screening platform for ADPKD (13) that employs *Pkd1*-null postnatal cells of proximal tubule origin, *Pkd1*-null mouse embryonic kidney cells of collecting duct origin (MEK-null), and *Pkd1*^−/−^ human kidney epithelial cells in order to evaluate a set of 8814 approved drugs and studied compounds principally having anti-neoplastic usage. It resulted in 155 compounds that reduced the viability of mutant cells but had minimal effects on wild-type cells. The investigators inferred that 109 of these compounds reduced cyst growth in *Pkd1*^−/−^ cells cultured in a 3D matrix. Furthermore, with the aim of screening out possible therapies that failed to be translated in the treatment of the human disease, the platform has been extended by introducing the evaluation of the compounds in a panel of primary ADPKD and control human kidney epithelial cells. This step allowed the repurposing of 18 compounds that affected the viability of ADPKD cells with minimal effects on control human kidney epithelial cells. These compounds are: (i) Briciclib, Prexasertib, AHPN, and IMD-0354 which have been not yet been analyzed in the context of ADPKD; (ii) Cytarabine, Ancitabine, Clofarabine, Gemcitabine, Adefovir, and Topotecan, Belotecan, and 9-Methoxycamptothecin, which inhibit the proliferation of *Pkd1*^−/−^ cells by directly interfering with DNA replication; (iii) Combretastatin A-4, Leptomycin B, Delanzomib, CB30865, Cerivastatin, and Pitavastatin which have been previously proposed as potential therapeutic options for ADPKD (142–144).

In short, given the complexity of the altered pathways in cyst-lining epithelia, and the availability of a broad range of potential targets, powerful strategies are needed to make faster advances in this field. Systems medicine and drug repurposing for the development of new treatments can help expedite the discovery of new effective treatments. The tools and the approaches presented in this section represent valuable platforms to repurpose chemical compounds and to identify drug targets for ADPKD treatment. All the presented studies discussed are of high relevance to ADPKD patients, as Tolvaptan, is the only approved therapy but has limited treatment options. The identified compounds represent a starting point for future efficacy studies employing relevant *ex vivo* and *in vivo* ADPKD models.

## Use of Omics Data and Future Therapeutics in ADPKD

Omics data are valuable tools for elucidating key genes and pathways associated with different aspects of ADPKD. However, one of the main drawbacks is the much of the profiling data from mouse models and patients has been published on public databases (the reader can find details about the most known datasets in Table 2), but it has not yet been exhaustively analyzed with the most appropriate computational approaches. Some points have to be carefully considered: (i) how samples are organized in different groups in order to follow a differential expression analysis; (ii) how results from a computational analysis are corrected for multiple comparisons in order to have strong statistics; and (iii) how to take into consideration that data from independent studies could be limited by the fact that they were derived from single cohort and by the samples heterogeneity.

**Table 2:** A list of datasets related to ADPKD.

**Database**	**Dataset series**	**Experiment type**	**Organism**	**References**
GEO	GSE35831	Expression profiling by array	Homo Sapiens	(145)
GEO	GSE7869	Expression profiling by array	Homo Sapiens	(17)
GEO	GSE9167	Expression profiling by array	Mus Musculus	(98)
GEO	GSE24352	Expression profiling by array	Mus Musculus	(95)
GEO	GSE32586	Expression profiling by array	Mus Musculus	(99)
GEO	GSE19460	Expression profiling by array	Rattus Norvegicus	(146)
GEO	GSE11500	Expression profiling by array	Rattus Norvegicus	(147)
GEO	GSE13065	Expression profiling by array	Rattus Norvegicus	(148)
GEO	GSE72554	Expression profiling by array	Mus Musculus	(10)
GEO	GSE86509	Expression profiling by high throughput sequencing Non-coding RNA profiling by high throughput sequencing	Mus Musculus	(94)
GEO	GSE121563	Expression profiling by array	Mus Musculus	(12)
GEO	GSE13452	Expression profiling by array	Mus Musculus	(149)
GEO	GSE2673	Expression profiling by array	Mus Musculus	(150)
GEO	GSE23079	Expression profiling by array	Rattus Norvegicus	(151)
GEO	GSE74451	Expression profiling by array	Homo Sapiens	(152)
GEO	GSE75578	Expression profiling by array	Rattus Norvegicus	(16)
GEO	GSE101308	Expression profiling by array	Homo Sapiens	(153)
GEO	GSE108864	Expression profiling by high throughput sequencing	Mus Musculus	(100)
GEO	GSE131277	Expression profiling by high throughput sequencing	Mus Musculus	(145)
GEO	GSE134721	Expression profiling by high throughput sequencing	Homo Sapiens Mus Musculus	(125)
GEO	GSE128524	Expression profiling by high throughput sequencing Genome binding/occupancy profiling by high throughput sequencing	Mus Musculus	(154)
GEO	GSE137945	Expression profiling by high throughput sequencing	Mus Musculus	(97)
GEO	GSE89764	Expression profiling by high throughput sequencing	Mus Musculus	(54)
GEO	GSE108864	Expression profiling by high throughput sequencing	Mus Musculus	(100)
GEO	GSE141281	Expression profiling by high throughput sequencing Genome binding/occupancy profiling by high throughput sequencing	Mus Musculus	(126)
PRIDE	PXD001075	Proteomics	Homo Sapiens	(155)
MetaboLights	MTBLS677	Metabolic Tracing Experiments	Mus Musculus	(12)
Podrini et al.	Suppl. Data	Metabolomics	Mus Musculus	(12)
Podrini et al.	Suppl. Data	Lipidomics	Mus Musculus	(12)
Menezes et al.	Suppl. Data	Quantile-normalized microRNA expression data	Mus Musculus	(95)
ArrayExpress	E-MTAB-8086	RNA-seq of coding RNA, compound treatment design, disease state design, genetic modification design	Mus Musculus	(14)
ArrayExpress	E-MTAB-4188	Transcription profiling by array, disease state design	Homo sapiens	(156)
ArrayExpress	E-MTAB-6640	RNA-seq of coding RNA, stimulus or stress design	Mus Musculus	(157)
ArrayExpress	E-MTAB-6641	RNA-seq of coding RNA, stimulus or stress design	Mus Musculus	(157)

*Details about the different databases can be found in the following papers: GEO (158), MetaboLights (159), ArrayExpress (160), and Pride (161)*.

It has also been observed that genome-scale profiling analysis can lack reproducibility because a large number of hypothesis tests are based on relatively small sample sizes. This problem can be solved by executing a meta-analysis based on the integration of different studies, which can increase statistical power and lead to more robust inferences (162, 163). However, the development of reliable tools to integrate different datasets remains a challenge due to low data comparability and consistency. A cross species meta-analysis was carried out by (164) with the aim of identifying conserved pathways that could be key targets for ADPKD therapy. Their analysis was rooted on mouse, rat, and human microarray datasets and revealed statistically significant enriched dysregulated pathways and processes conserved across all the species. Processes related to genomic instability, growth factors and hormones, protein localization in ER and insulin resistance promote cysts formation that is regulated by key pathways mediating apoptosis, cell proliferation, cell adhesion, and differentiation. In contrast, cysts progression seems to be a consequence of processes associated with cell adhesion and inflammation. In addition, a causative link among immune response pathways, signaling pathways, and cellular architecture pathways seems to affect cyst development.

Regarding the drug repositioning for the ADPKD, we would like to highlight that treatments and their targets are biased toward the most studied proteins and disorders. Future functional wet-experiments are needed to infer the actual contribution of each gene involved in cyst enlargement and disease progression. Further investigations will be facilitated by the development and the continuous update of the molecules database with drug-like properties.

Of note, there is a lack of knowledge about specific biomarkers, particularly in ADPKD pediatric patients. A recent work tried to identify plasma fingerprints in children and young adult patients with preserved kidney functions (67). The goal of the study was to identify markers and associated pathways involved in the progression of pediatric ADPKD over 36 months. The authors collected plasma samples from 58 young patients during a phase III clinical trial designed to test the effectiveness of pravastatin on slowing down cyst progression. In this study, metabolomics analysis revealed that metabolites deriving from the TCA cycle were associated with ADPKD progression. This is a set example of how metabolomics can offer a powerful tool for the prediction of the patient outcomes in clinical trials. However, there are also limited therapies focusing on treating ADPKD in early childhood where the benefits could be more likely. Screening of at-risk children is controversial and not recommended (1, 165) because of its potential to cause psychological harm. There is not yet a means of stratifying patients by rapid progressors of disease or not. To achieve this, it will be fundamental to start lifelong therapy at a later stage. The main burden of ADPKD is that it does not occur until adulthood and some children are usually asymptomatic. This may, as a consequence, influence their life-style. There is a significant discrepancy regarding data obtained in adults vs. children as diagnostic criteria are highly variable, leading to incomparable studies and results (166). In children with ADPKD, EU guidelines recommends to stratify high risk patients and Tolvaptan is currently being tested in EU in children with ADPKD with high risk of rapid progression (167). The result of this study could form the basis for understanding the tolerability of tolvaptan in patients who still have a good renal function and not yet compromised. What remains to be evaluated is whether the treatment could be effective if initiated at an early stage, before cysts are formed, while the kidneys are still well-preserved.

Several effective therapies are under current investigation. It is unclear why defective PKD proteins alter signaling pathways involved in bio-energetics processes. Despite the gap of knowledge, current therapeutic interventions and clinical trials are ongoing with promising results. It is still debated whether the best strategy is to target one single pathway or to target multiple pathways simultaneously. In the future, an increase in the application of less invasive therapies, such as dietary restrictions, may be proposed to patients because, as described above, certain dietary interventions have been successful in slowing the progression of ADPKD in animal models. For example, time-restricted feeding improved kidney function and reduced cytogenesis, and a ketogenic diet also demonstrated similar beneficial effects in ameliorating cytogenesis (39). Human trials are ongoing and this will be a stepping stone to pursue non-invasive therapies which can be exploited and refined with a customized diet. Although the results seem promising, the benefits of the PKD ketogenic diet in human are unpredictable and the risk of potential side effects, such as cardiovascular problems or impaired liver function, is unknown. It is known that nutritional aspects such as high fluid and low sodium diet are beneficial for improving cysts formation and are also able to restrict phosphorus and protein intake (168); however, this intervention is not prescribed in the presence of a severe renal function. At present, perhaps the important aspect to consider would be to follow a balanced diet and to avoid a large amount of refined carbohydrates. Further impact studies are needed on this issue as to address the benefits of non-invasive therapies in ADPKD.

## Author Contributions

CP and RP wrote the manuscript, reviewed and edited the manuscript before submission. Both authors contributed to the article and approved the submitted version.

## Funding

The work on metabolism and PKD was funded by the Italian Ministry of Health (GR-2016-02364851 to CP and RP).

## Conflict of Interest

CP is a co-inventor on the use of metabolic inhibitors in Polycystic Kidney Disease. The remaining author declares that the research was conducted in the absence of any commercial or financial relationships that could be construed as a potential conflict of interest.

## Publisher's Note

All claims expressed in this article are solely those of the authors and do not necessarily represent those of their affiliated organizations, or those of the publisher, the editors and the reviewers. Any product that may be evaluated in this article, or claim that may be made by its manufacturer, is not guaranteed or endorsed by the publisher.

## References

[1] Chapman AB Devuyst O Eckardt KU Gansevoort RT Harris T Horie S. Autosomal-dominant polycystic kidney disease (ADPKD): executive summary from a Kidney Disease: Improving Global Outcomes (KDIGO) Controversies Conference *Kidney Int*. (2015) 88:17–27. 10.1038/ki.2015.59 25786098 PMC4913350

[2] Porath B Gainullin VG Cornec-Le Gall E Dillinger EK Heyer CM Hopp K. Mutations in GANAB, Encoding the Glucosidase IIalpha Subunit, Cause Autosomal-Dominant Polycystic Kidney and Liver Disease. Am J Hum Genet. (2016) 98:1193–207. 10.1016/j.ajhg.2016.05.004 27259053 PMC4908191

[3] Sandford R Sgotto B Aparicio S Brenner S Vaudin M Wilson RK. Comparative analysis of the polycystic kidney disease 1 (PKD1) gene reveals an integral membrane glycoprotein with multiple evolutionary conserved domains. Hum Mol Genet. (1997) 6:1483–9. 10.1093/hmg/6.9.1483 9285785

[4] Su Q Hu F Ge X Lei J Yu S Wang T. Structure of the human PKD1-PKD2 complex. Science. (2018) 361:eaat9819. 10.1126./science.aat9819 30093605

[5] Qian F Germino FJ Cai Y Zhang X Somlo S Germino GG. PKD1 interacts with PKD2 through a probable coiled-coil domain. Nat Genet. (1997) 16:179–83. 10.1038/ng0697-179 9171830

[6] Nauli SM Alenghat FJ Luo Y Williams E Vassilev P Elia L. Polycystins 1 and 2 mediate mechanosensation in the primary cilium of kidney cells. Nat Genet. (2003) 33:129–37. 10.1038/ng1076 12514735

[7] Delling M Indzhykulian AA Liu X Li Y Xie T Corey DP. Primary cilia are not calcium-responsive mechanosensors. Nature. (2016) 531:656–60. 10.1038/nature17426 27007841 PMC4851444

[8] Bergmann C Guay-Woodford LM Harris PC Horie S Peters DJM Torres VE. Polycystic kidney disease. Nat Rev Dis Primers. (2018) 4:50. 10.1038/s41572-018-0047-y 30523303 PMC6592047

[9] Rowe I Chiaravalli M Mannella V Ulisse V Quilici G Pema M. Defective glucose metabolism in polycystic kidney disease identifies a new therapeutic strategy. Nat Med. (2013) 19:488–93. 10.1038/nm.3092 23524344 PMC4944011

[10] Menezes LF Lin CC Zhou F Germino GG. Fatty acid oxidation is impaired in an orthologous mouse model of autosomal dominant polycystic kidney disease. Ebiomedicine. (2016) 5:183–192. 10.1016/j.ebiom.2016.01.027 27077126 PMC4816756

[11] Padovano V Kuo IY Stavola LK Aerni HR Flaherty BJ Chapin HC. The polycystins are modulated by cellular oxygen-sensing pathways and regulate mitochondrial function. Mol Biol Cell. (2017) 28:261–9. 10.1091/mbc.e16-08-0597 27881662 PMC5231895

[12] Podrini C Rowe I Pagliarini R Costa ASH Chiaravalli M Meo D. Dissection of metabolic reprogramming in polycystic kidney disease reveals coordinated rewiring of bioenergetic pathways. Commun Biol. (2018) 1:194. 10.1038/s42003-018-0200-x 30480096 PMC6240072

[13] Asawa RR Danchik C Zahkarov A Chen Y Voss T Jadhav A. A high-throughput screening platform for Polycystic Kidney Disease (PKD) drug repurposing utilizing murine and human ADPKD cells. Sci Rep. (2020) 10:4203. 10.1038/s41598-020-61082-3 32144367 PMC7060218

[14] Malas TB Leonhard WN Bange H Granchi Z Hettne KM Van Westen GJP. Prioritization of novel ADPKD drug candidates from disease-stage specific gene expression profiles. EBioMed. (2020) 51:102585. 10.1016/j.ebiom.2019.11.046 31879244 PMC7000333

[15] Chen L Zhou X Fan LX Yao Y Swenson-Fields KI Gadjeva M. Macrophage migration inhibitory factor promotes cyst growth in polycystic kidney disease. J Clin Invest. (2015) 125:2399–412. 10.1172/JCI80467 25961459 PMC4497763

[16] Riwanto M Kapoor S Rodriguez D Edenhofer I Segerer S Wuthrich RP. Inhibition of aerobic glycolysis attenuates disease progression in polycystic kidney disease. PLoS ONE. (2016) 11:e0146654. 10.1371/journal.pone.0146654 26752072 PMC4708993

[17] Song X Giovanni Di V He N Wang K Ingram A Rosenblum ND. Systems biology of autosomal dominant polycystic kidney disease (ADPKD): computational identification of gene expression pathways and integrated regulatory networks. Hum Mol Genet. (2009) 18:2328–43. 10.1093/hmg/ddp165 19346236

[18] Hallows KR Althouse AD Li B Saitta B Abebe KZ Bae KT. Association of baseline urinary metabolic biomarkers with ADPKD severity in TAME-PKD clinical trial participants. Kidney. (2021) 2:795–808. 10.34067/KID.0005962020 34316721 PMC8312696

[19] Seliger SL Abebe KZ Hallows KR Miskulin DC Perrone RD Watnick T. A randomized clinical trial of metformin to treat autosomal dominant polycystic kidney disease. Am J Nephrol. (2018) 47:352–60. 10.1159/000488807 29779024 PMC6010317

[20] Schrier RW. Optimal care of autosomal dominant polycystic kidney disease patients. Nephrology. (2006) 11:124–30. 10.1111/j.1440-1797.2006.00535.x 16669974

[21] Shamshirsaz AA Reza Bekheirnia M Kamgar M Johnson AM McFann K Cadnapaphornchai M. Autosomal-dominant polycystic kidney disease in infancy and childhood: progression and outcome. Kidney Int. (2005) 68:2218–24. 10.1111/j.1523-1755.2005.00678.x 16221221

[22] Taylor M Johnson AM Tison M Fain P Schrier RW. Earlier diagnosis of autosomal dominant polycystic kidney disease: importance of family history and implications for cardiovascular and renal complications. Am J Kidney Dis. (2005) 46:415–23. 10.1053/j.ajkd.2005.05.029 16129202

[23] Cadnapaphornchai MA Fick-Brosnahan GM Duley I Johnson AM Strain JD DeGroff CG. Design and baseline characteristics of participants in the study of antihypertensive therapy in children and adolescents with autosomal dominant polycystic kidney disease (ADPKD). Contemp Clin Trials. (2005) 26:211–22. 10.1016/j.cct.2005.01.001 15837441

[24] Fick-Brosnahan GM Belz MM McFann KK Johnson AM Schrier RW. Relationship between renal volume growth and renal function in autosomal dominant polycystic kidney disease: a longitudinal study. Am J Kidney Dis. (2002) 39:1127–34. 10.1053/ajkd.2002.33379 12046022

[25] Kelleher CL McFann KK Johnson AM Schrier RW. Characteristics of hypertension in young adults with autosomal dominant polycystic kidney disease compared with the general U.S. population. Am J Hypertens. (2004) 17:1029–34. 10.1016/j.amjhyper.2004.06.020 15533729

[26] Cadnapaphornchai MA George DM McFann K Wang W Gitomer B Strain JD. Effect of pravastatin on total kidney volume, left ventricular mass index, and microalbuminuria in pediatric autosomal dominant polycystic kidney disease. Clin J Am Soc Nephrol. (2014) 9:889–96. 10.2215/CJN.08350813 24721893 PMC4011448

[27] Kim K Trott JF Gao G Chapman A Weiss RH. Plasma metabolites and lipids associate with kidney function and kidney volume in hypertensive ADPKD patients early in the disease course. BMC Nephrol. (2019) 20:66. 10.1186/s12882-019-1249-6 30803434 PMC6388487

[28] Schrier RW Abebe KZ Perrone RD Torres VE Braun WE Steinman TI. Blood pressure in early autosomal dominant polycystic kidney disease. N Engl J Med. (2014) 371:2255–66. 10.1056/NEJMoa1402685 25399733 PMC4343258

[29] Hogan MC Abebe K Torres VE Chapman AB Bae KT Tao C. Liver involvement in early autosomal-dominant polycystic kidney disease. Clin Gastroenterol Hepatol. (2015) 13:155–64. e6. 10.1016/j.cgh.2014.07.051 25111236 PMC4267913

[30] Torres VE Chapman AB Perrone RD Bae KT Abebe KZ Bost JE. Analysis of baseline parameters in the HALT polycystic kidney disease trials. Kidney Int. (2012) 81:577–85. 10.1038/ki.2011.411 22205355 PMC3580956

[31] Torres VE Abebe KZ Chapman AB Schrier RW Braun WE Steinman TI. Angiotensin blockade in late autosomal dominant polycystic kidney disease. N Engl J Med. (2014) 371:2267–76. 10.1056/NEJMoa1402686 25399731 PMC4284824

[32] Ruggenenti P Gentile G Perico N Perna A Barcella L Trillini M. Effect of sirolimus on disease progression in patients with autosomal dominant polycystic kidney disease and CKD stages 3b-4. Clin J Am Soc Nephrol. (2016) 11:785–94. 10.2215/CJN.09900915 26912555 PMC4858487

[33] Wahl PR Serra AL Le Hir M Molle KD Hall MN Wuthrich RP. Inhibition of mTOR with sirolimus slows disease progression in Han:SPRD rats with autosomal dominant polycystic kidney disease (ADPKD). Nephrol Dial Transplant. (2006) 21:598–604. 10.1093/ndt/gfi181 16221708

[34] Serra AL Poster D Kistler AD Krauer F Raina S Young J. Sirolimus and kidney growth in autosomal dominant polycystic kidney disease. N Engl J Med. (2010) 363:820–9. 10.1056/NEJMoa0907419 20581391

[35] Braun M Young J Reiner CS Poster D Wuthrich RP Serra AL. Ovarian toxicity from sirolimus. N Engl J Med. (2012) 366:1062–4. 10.1056/NEJMc1113145 22417271

[36] Braun M Young J Reiner CS Poster D Krauer F Kistler AD. Low-dose oral sirolimus and the risk of menstrual-cycle disturbances and ovarian cysts: analysis of the randomized controlled SUISSE ADPKD trial. PLoS ONE. (2012) 7:e45868. 10.1371/journal.pone.0045868 23071528 PMC3468602

[37] Serra AL Kistler AD Poster D Struker M Wuthrich RP Weishaupt D. Clinical proof-of-concept trial to assess the therapeutic effect of sirolimus in patients with autosomal dominant polycystic kidney disease: SUISSE ADPKD study. BMC Nephrol. (2007) 8:13. 10.1186/1471-2369-8-13 17868472 PMC2048941

[38] Walz G Budde K Mannaa M Nurnberger J Wanner C Sommerer C. Everolimus in patients with autosomal dominant polycystic kidney disease. N Engl J Med. (2010) 363:830–40. 10.1056/NEJMoa1003491 20581392

[39] Torres JA Kruger SL Broderick C Amarlkhagva T Agrawal S Dodam JR. Ketosis ameliorates renal cyst growth in polycystic kidney disease. Cell Metab. (2019) 30:1007–23.e5. 10.1016/j.cmet.2019.09.012 31631001 PMC6904245

[40] Gattone VH II Wang X Harris PC Torres VE. Inhibition of renal cystic disease development and progression by a vasopressin V2 receptor antagonist. Nat Med. (2003) 9:1323–6. 10.1038/nm935 14502283

[41] Torres VE Wang X Qian Q Somlo S Harris PC Gattone VH II. Effective treatment of an orthologous model of autosomal dominant polycystic kidney disease. Nat Med. (2004) 10:363–4. 10.1038/nm1004 14991049

[42] Watkins PB Lewis JH Kaplowitz N Alpers DH Blais JD Smotzer DM. Clinical pattern of tolvaptan-associated liver injury in subjects with autosomal dominant polycystic kidney disease: analysis of clinical trials database. Drug Saf. (2015) 38:1103–13. 10.1007/s40264-015-0327-3 26188764 PMC4608984

[43] Torres VE Gansevoort RT Czerwiec FS. Tolvaptan in autosomal dominant polycystic kidney disease. N Engl J Med. (2013) 368:1259. 10.1056/NEJMc1300762 23534568

[44] Torres VE Meijer E Bae KT Chapman AB Devuyst O Gansevoort RT. Rationale and design of the TEMPO (Tolvaptan Efficacy and Safety in Management of Autosomal Dominant Polycystic Kidney Disease and its Outcomes) 3-4 Study. Am J Kidney Dis. (2011) 5:692–9. 10.1053/j.ajkd.2010.11.029 21333426

[45] Kher A. Tolvaptan in autosomal dominant polycystic kidney disease. N Engl J Med. (2013) 368:1257–8. 10.1056/NEJMc1300762 23534570

[46] Spital A. Tolvaptan in autosomal dominant polycystic kidney disease. N Engl J Med. (2013) 368:1257. 10.1056/NEJMc1300762 23534569

[47] Jouret F Krzesinski JM. Tolvaptan in autosomal dominant polycystic kidney disease. N Engl J Med. (2013) 368:1258–9. 10.1056/NEJMc1300762 23534572

[48] Sexton DJ. Tolvaptan in autosomal dominant polycystic kidney disease. N Engl J Med. (2013) 368:1258. 10.1056/NEJMc1300762 23534571

[49] Vander Heiden MG DeBerardinis RJ. Understanding the Intersections between Metabolism and Cancer Biology. Cell. (2017) 168:657–69. 10.1016/j.cell.2016.12.039 28187287 PMC5329766

[50] Kuo IY Brill AL Lemos FO Jiang JY Falcone JL Kimmerling EP. Polycystin 2 regulates mitochondrial Ca(2+) signaling, bioenergetics, and dynamics through mitofusin 2. Sci Signal. (2019) 12:aat7397. 10.1126/scisignal.aat7397 31064883 PMC6855602

[51] Lin CC Kurashige M Liu Y Terabayashi T Ishimoto Y Wang T. A cleavage product of Polycystin-1 is a mitochondrial matrix protein that affects mitochondria morphology and function when heterologously expressed. Sci Rep. (2018) 8:2743. 10.1038/s41598-018-20856-6 29426897 PMC5807443

[52] Ishimoto Y Inagi R Yoshihara D Kugita M Nagao S Shimizu A. Mitochondrial abnormality facilitates cyst formation in autosomal dominant polycystic kidney disease. Mol Cell Biol. (2017) 37:e00337–17. 10.1128/MCB.00337-17 28993480 PMC5705822

[53] Cassina L Chiaravalli M Boletta A. Increased mitochondrial fragmentation in polycystic kidney disease acts as a modifier of disease progression. FASEB J. (2020) 34:6493–507. 10.1096/fj.201901739RR 32239723

[54] Hajarnis S Lakhia R Yheskel M Williams D Sorourian M Liu X. microRNA-17 family promotes polycystic kidney disease progression through modulation of mitochondrial metabolism. Nat Commun. (2017) 8:14395. 10.1038/ncomms14395 28205547 PMC5316862

[55] Blazer-Yost BL Haydon J Eggleston-Gulyas T Chen JH Wang X Gattone V. Pioglitazone attenuates cystic burden in the PCK rodent model of polycystic kidney disease. PPAR Res. (2010) 2010:274376. 10.1155/2010/274376 21052534 PMC2968120

[56] Yoshihara D Kurahashi H Morita M Kugita M Hiki Y Aukema HM. PPAR-gamma agonist ameliorates kidney and liver disease in an orthologous rat model of human autosomal recessive polycystic kidney disease. Am J Physiol Renal Physiol. (2011) 300:F465–74. 10.1152/ajprenal.00460.2010 21147840 PMC3044004

[57] Dai B Liu Y Mei C Fu L Xiong X Zhang Y. Rosiglitazone attenuates development of polycystic kidney disease and prolongs survival in Han:SPRD rats. Clin Sci. (2010) 119:323–33. 10.1042/CS20100113 20507283

[58] Ma Y Temkin SM Hawkridge AM Guo C Wang W Wang XY. Fatty acid oxidation: an emerging facet of metabolic transformation in cancer. Cancer Lett. (2018) 435:92–100. 10.1016/j.canlet.2018.08.006 30102953 PMC6240910

[59] Hensley CT Wasti AT DeBerardinis RJ. Glutamine and cancer: cell biology, physiology, and clinical opportunities. J Clin Invest. (2013) 123:3678–3684. 10.1172/JCI69600 23999442 PMC3754270

[60] DeBerardinis RJ Mancuso A Daikhin E Nissim I Yudkoff M Wehrli S. Beyond aerobic glycolysis: transformed cells can engage in glutamine metabolism that exceeds the requirement for protein and nucleotide synthesis. Proc Natl Acad Sci USA. (2007) 104:19345–50. 10.1073/pnas.0709747104 18032601 PMC2148292

[61] Hwang VJ Kim J Rand A Yang C Sturdivant S Hammock B. The cpk model of recessive PKD shows glutamine dependence associated with the production of the oncometabolite 2-hydroxyglutarate. Am J Physiol Renal Physiol. (2015) 309:F492–8. 10.1152/ajprenal.00238.2015 26155843 PMC4572393

[62] Shim EH Livi CB Rakheja D Tan J Benson D Parekh V. L-2-Hydroxyglutarate: an epigenetic modifier and putative oncometabolite in renal cancer. Cancer Discov. (2014) 4:1290–8. 10.1158/2159-8290.CD-13-0696 25182153 PMC4286872

[63] Seeger-Nukpezah T Geynisman DM Nikonova AS Benzing T Golemis EA. The hallmarks of cancer: relevance to the pathogenesis of polycystic kidney disease. Nat Rev Nephrol. (2015) 11:515–34. 10.1038/nrneph.2015.46 25870008 PMC5902186

[64] Soomro I Sun Y Diggs Li Z Hatzivassiliou L Thomas G. Glutamine metabolism via glutaminase 1 in autosomal-dominant polycystic kidney disease. Nephrol Dial Transplant. (2018) 33:1343–53. 10.1093/ndt/gfx349 29420817 PMC6070111

[65] Lomelino CL Andring JT McKenna R Kilberg MS. Asparagine synthetase: function, structure, and role in disease. J Biol Chem. (2017) 292:19952–8. 10.1074/jbc.R117.819060 29084849 PMC5723983

[66] Zhang J Fan J Venneti S Cross JR Takagi T Bhinder B. Asparagine plays a critical role in regulating cellular adaptation to glutamine depletion. Mol Cell. (2014) 56:205–18. 10.1016/j.molcel.2014.08.018 25242145 PMC4224619

[67] Baliga MM Klawitter J Christians U Hopp K Chonchol M Gitomer BY. Metabolic profiling in children and young adults with autosomal dominant polycystic kidney disease. Sci Rep. (2021) 11:6629. 10.1038/s41598-021-84609-8 33758231 PMC7988179

[68] Trott JF Hwang VJ Ishimaru T Chmiel KJ Zhou JX Shim K. Arginine reprogramming in ADPKD results in arginine-dependent cystogenesis. Am J Physiol Renal Physiol. (2018) 315:F1855–68. 10.1152/ajprenal.00025.2018 30280600 PMC6336982

[69] Patil MD Bhaumik J Babykutty S Banerjee UC Fukumura D. Arginine dependence of tumor cells: targeting a chink in cancer's armor. Oncogene. (2016) 35:4957–72. 10.1038/onc.2016.37 27109103 PMC5457742

[70] Wise DR Thompson CB. Glutamine addiction: a new therapeutic target in cancer. Trends Biochem Sci. (2010) 35:427–33. 10.1016/j.tibs.2010.05.003 20570523 PMC2917518

[71] Flowers EM Sudderth J Zacharias L Mernaugh G Zent R DeBerardinis RJ. Lkb1 deficiency confers glutamine dependency in polycystic kidney disease. Nat Commun. (2018) 9:814. 10.1038/s41467-018-03036-y 29483507 PMC5827653

[72] Kahveci AS Barnatan TT Kahveci A Adrian AE Arroyo J Eirin A. Oxidative stress and mitochondrial abnormalities contribute to decreased endothelial nitric oxide synthase expression and renal disease progression in early experimental polycystic kidney disease. Int J Mol Sci. (2020) 21:1194. 10.3390/ijms21061994 32183375 PMC7139316

[73] Nowak KL Farmer H Cadnapaphornchai MA Gitomer B Chonchol M. Vascular dysfunction in children and young adults with autosomal dominant polycystic kidney disease. Nephrol Dial Transplant. (2017) 32:342–7. 10.1093/ndt/gfw013 28186577 PMC5837416

[74] Klawitter J Reed-Gitomer BY McFann K Pennington A Klawitter J Abebe KZ. Endothelial dysfunction and oxidative stress in polycystic kidney disease. Am J Physiol Renal Physiol. (2014) 307:F1198–206. 10.1152/ajprenal.00327.2014 25234311 PMC4254971

[75] Cyr AR Huckaby LV Shiva SS Zuckerbraun BS. Nitric oxide and endothelial dysfunction. Crit Care Clin. (2020) 36:307–21. 10.1016/j.ccc.2019.12.009 32172815 PMC9015729

[76] Verdoodt A Honore PM Jacobs R De Waele HD. Do statins induce or protect from acute kidney injury and chronic kidney disease: an update review in 2018. J Transl Int Med. (2018) 6:21–5. 10.2478/jtim-2018-0005 29607300 PMC5874483

[77] Zheng Z Jayaram R Jiang L Emberson J Zhao Y Du L. Perioperative rosuvastatin in cardiac surgery. N Engl J Med. (2016) 374:1744–53. 10.1056/NEJMoa1507750 27144849

[78] Klawitter J McFann K Pennington AT Wang W Klawitter J Christians U. Pravastatin therapy and biomarker changes in children and young adults with autosomal dominant polycystic kidney disease. Clin J Am Soc Nephrol. (2015) 10:1534–41. 10.2215/CJN.11331114 26224879 PMC4559520

[79] Brosnahan GM Abebe KZ Rahbari-Oskoui FF Patterson CG Bae KT Schrier RW. Effect of statin therapy on the progression of autosomal dominant polycystic kidney disease. A secondary analysis of the HALT PKD trials. Curr Hypertens Rev. (2017) 13:109–20. 10.2174/1573402113666170427142815 28460625 PMC5688015

[80] Steuer R Gross T Selbig J Blasius B. Structural kinetic modeling of metabolic networks. Proc Natl Acad Sci USA. (2006) 103:11868–73. 10.1073/pnas.0600013103 16880395 PMC1524928

[81] Saa PA Nielsen LK. Formulation, construction and analysis of kinetic models of metabolism: A review of modelling frameworks. Biotechnol Adv. (2017) 35:981–1003. 10.1016/j.biotechadv.09005 28916392

[82] Keizer J. Statistical Thermodynamics of Nonequilibrium Processeì. New York, NY; Berlin; Heidelberg; London; Paris; Tokyo: Springeí Verlag (1989).

[83] Michaelis L Menten M Johnson KA Goody R. The original Michaelis constant: translation of the 1913 Michaelis-Menten paper. Biochemistry. (2011) 50:8264–9. 10.1021/bi201284u 21888353 PMC3381512

[84] Cornish-Bowden A. Fundamentals of Enzyme Kinetics (1979).

[85] Savageau MA. Biochemical systems analysis. I Some mathematical properties of the rate law for the component enzymatic reactions. J Theor Biol. (1969) 25:365–9. 10.1016/s0022-5193(69)80026-3 5387046

[86] Orth JD Thiele I Palsson BO. What is flux balance analysis? Nat Biotechnol. (2010) 28:245–8. 10.1038/nbt.1614 20212490 PMC3108565

[87] Pagliarini R Castello R Napolitano F Borzone R Annunziata P Mandrile G. *In silico* modeling of liver metabolism in a human disease reveals a key enzyme for histidine and histamine homeostasis. Cell Rep. (2016) 15:2292–300. 10.1016/j.celrep.2016.05.014 27239044 PMC4906368

[88] Pagliarini R di Bernardo D. A genome-scale modeling approach to study inborn errors of liver metabolism: toward an in silico patient. J Comput Biol. (2016). 20:383–97. 10.1089/cmb.2012.0276 23464878 PMC3646339

[89] Pagliarini R Boletta A. *In SILICO* simulations predict a causative link between increased glycolysis and metabolic reprogramming in autosomal dominant polycystic kidney disease. In: 2019 IEEE Conference on Computational Intelligence in Bioinformatics and Computational Biology (Siena). (2019). p. 1–9. 10.1109/CIBCB.2019.8791243

[90] Rahimmanesh I Fatehi R. Systems biology approaches toward autosomal dominant polycystic kidney disease (ADPKD). Clin Transl Med. (2020) 9:1. 10.1186/s40169-019-0254-5 31907669 PMC6944722

[91] Kocyigit I Eroglu E Kaynar AS Kocer D Kargi S Zararsiz G. The association of endothelin-1 levels with renal survival in polycystic kidney disease patients. J Nephrol. (2019) 32:83–91. 10.1007/s40620-018-0514-2 30022320

[92] Chang MY Ong AC. M. Endothelin in polycystic kidney disease. Contrib Nephrol. (2011) 172:200–9. 10.1159/000328701 21894000

[93] Liu D Huo Y Chen S Xu D Yang B Xue C. Identification of key genes and candidated pathways in human autosomal dominant polycystic kidney disease by bioinformatics analysis. Kidney Blood Press Res. (2019) 44:533–52. 10.1159/000500458 31330507

[94] Woo YM Kim DY Koo NJ Kim YM Lee S Ko JY. Profiling of miRNAs and target genes related to cystogenesis in ADPKD mouse models. Sci Rep. (2017) 7:14151. 10.1038/s41598-017-14083-8 29074972 PMC5658336

[95] Pandey P Qin S Ho J Zhou J Kreidberg JA. Systems biology approach to identify transcriptome reprogramming and candidate microRNA targets during the progression of polycystic kidney disease. BMC Syst Biol. (2011) 5:56. 10.1186/1752-0509-5-56 21518438 PMC3111376

[96] Patel V Williams D Hajarnis S Hunter R Pontoglio M Somlo S. miR-17~92 miRNA cluster promotes kidney cyst growth in polycystic kidney disease. Proc Natl Acad Sci USA. (2013) 110:10765–70. 10.1073/pnas.1301693110 23759744 PMC3696812

[97] Lakhia R Yheskel M Flaten A Ramalingam H Aboudehen K Ferre S. Interstitial microRNA miR-214 attenuates inflammation and polycystic kidney disease progression. JCI Insight. (2020) 5:133785. 10.1172/jci.insight.133785 32182218 PMC7205276

[98] Piontek K Menezes LF Garcia-Gonzalez MA Huso DL Germino GG. A critical developmental switch defines the kinetics of kidney cyst formation after loss of Pkd1. Nat Med. (2007) 13:1490–5. 10.1038/nm1675 17965720 PMC2302790

[99] Menezes LF Zhou F Patterson AD Piontek KB Krausz KW Gonzalez FJ. Network analysis of a Pkd1-mouse model of autosomal dominant polycystic kidney disease identifies HNF4alpha as a disease modifier. PLoS Genet. (2012) 8:e1003053. 10.1371/journal.pgen.1003053 23209428 PMC3510057

[100] Aboudehen K Farahani S Kanchwala M Chan SC Avdulov S Mickelson A. Long noncoding RNA Hoxb3os is dysregulated in autosomal dominant polycystic kidney disease and regulates mTOR signaling. J Biol Chem. (2018) 293:9388–98. 10.1074/jbc.RA118.001723 29716997 PMC6005429

[101] Lantinga-van Leeuwen IS Dauwerse JG Baelde HJ Leonhard WN van de Wal A Ward CJ. Lowering of Pkd1 expression is sufficient to cause polycystic kidney disease. Hum Mol Genet. (2004) 13:3069–77. 10.1093/hmg/ddh336 15496422

[102] Rossetti S Kubly VJ Consugar MB Hopp K Roy S Horsley SW. Incompletely penetrant PKD1 alleles suggest a role for gene dosage in cyst initiation in polycystic kidney disease. Kidney Int. (2009) 75:848–55. 10.1038/ki.2008.686 19165178 PMC2813773

[103] Hopp K Ward CJ Hommerding CJ Nasr SH Tuan HF Gainullin VG. Functional polycystin-1 dosage governs autosomal dominant polycystic kidney disease severity. J Clin Invest. (2012) 122:4257–73. 10.1172/JCI64313 23064367 PMC3484456

[104] Harris PC Torres VE. Genetic mechanisms and signaling pathways in autosomal dominant polycystic kidney disease. J Clin Invest. (2014) 124:2315–24. 10.1172/JCI72272 24892705 PMC4089452

[105] Torres VE Chapman AB Devuyst O Gansevoort RT Grantham JJ Higashihara E. Tolvaptan in patients with autosomal dominant polycystic kidney disease. N Engl J Med. (2012) 367:2407–18. 10.1056/NEJMoa1205511 23121377 PMC3760207

[106] Torres VE Chapman AB Devuyst O Gansevoort RT Perrone RD Koch G. Tolvaptan in later-stage autosomal dominant polycystic kidney disease. N Engl J Med. (2017) 377:1930–42. 10.1056/NEJMoa1710030 29105594

[107] Lian X Zhao J Wu X Zhang Y Li Q Lin S. The changes in glucose metabolism and cell proliferation in the kidneys of polycystic kidney disease mini-pig models. Biochem Biophys Res Commun. (2017) 488:374–81. 10.1016/j.bbrc.2017.05.060 28501615

[108] Distefano G Boca M Rowe I Wodarczyk C Ma L Piontek KB. Polycystin-1 regulates extracellular signal-regulated kinase-dependent phosphorylation of tuberin to control cell size through mTOR and its downstream effectors S6K and 4EBP1. Mol Cell Biol. (2009) 29:2359–71. 10.1128/MCB.01259-08 19255143 PMC2668371

[109] Shillingford JM Piontek KB Germino GG Weimbs T. Rapamycin ameliorates PKD resulting from conditional inactivation of Pkd1. J Am Soc Nephrol. (2010) 21:489–97. 10.1681/ASN.2009040421 20075061 PMC2831854

[110] Shibazaki S Yu Z Nishio S Tian X Thomson RB Mitobe M. Cyst formation and activation of the extracellular regulated kinase pathway after kidney specific inactivation of Pkd1. Hum Mol Genet. (2008) 17:1505–16. 10.1093/hmg/ddn039 18263604 PMC2902289

[111] Myint TM Rangan GK Webster AC. Treatments to slow progression of autosomal dominant polycystic kidney disease: systematic review and meta-analysis of randomized trials. Nephrology. (2014) 19:217–26. 10.1111/nep.12211 24460701

[112] Dirksen ML Crouch RJ. Selective inhibition of RNase H by dextran. J Biol Chem. (1981) 256:11569–73. 6170634

[113] Crooke ST. Molecular mechanisms of antisense oligonucleotides. Nucleic Acid Ther. (2017) 27:70–7. 10.1089/nat.2016.0656 28080221 PMC5372764

[114] Crooke ST Witztum JL Bennett CF Baker BF. RNA-targeted therapeutics. Cell Metab. (2018) 27:714–39. 10.1016/j.cmet.2018.03.004 29617640

[115] Crooke ST Liang XH Baker BF Crooke RM. Antisense technology: a review. J Biol Chem. (2021) 296:100416. 10.1016/j.jbc.2021.100416 33600796 PMC8005817

[116] Ravichandran K Zafar I He Z Doctor RB Moldovan R Mullick AE. An mTOR anti-sense oligonucleotide decreases polycystic kidney disease in mice with a targeted mutation in Pkd2. Hum Mol Genet. (2014) 23:4919–31. 10.1093/hmg/ddu208 24847003 PMC4140469

[117] Nowak KL Wang W Farmer-Bailey H Gitomer B Malaczewski M Klawitter J. Vascular dysfunction, oxidative stress, and inflammation in autosomal dominant polycystic kidney disease. Clin J Am Soc Nephrol. (2018) 13:1493–501. 10.2215/CJN.05850518 30228110 PMC6218833

[118] Klawitter J Klawitter J McFann K Pennington AT Abebe KZ Brosnahan G. Bioactive lipid mediators in polycystic kidney disease. J Lipid Res. (2014) 55:1139–49. 10.1194/jlr.P042176 24343898 PMC4031945

[119] Culliford A Phagura N Sharif A. Autosomal dominant polycystic kidney disease is a risk factor for posttransplantation diabetes mellitus: an updated systematic review and meta-analysis. Transplant Direct. (2020) 6:e553. 10.1097/TXD.0000000000000989 32548247 PMC7213605

[120] Ellison DH Ingelfinger JR. A quest–halting the progression of autosomal dominant polycystic kidney disease. N Engl J Med. (2014) 371:2329–31. 10.1056/NEJMe1412586 25399732

[121] Saigusa T Dang Y Mullick AE Yeh ST Zile MR Baicu CF. Suppressing angiotensinogen synthesis attenuates kidney cyst formation in a Pkd1 mouse model. FASEB J. (2016) 30:370–9. 10.1096/fj.15-279299 26391272 PMC4684522

[122] Fitzgibbon WR Dang Y Bunni MA Baicu CF Zile MR Mullick AE. Attenuation of accelerated renal cystogenesis in Pkd1 mice by renin-angiotensin system blockade. Am J Physiol Renal Physiol. (2018) 314:F210–8. 10.1152/ajprenal.00389.2017 29021226 PMC5866454

[123] Ravichandran K Ozkok A Wang Q Mullick AE Edelstein CL. Antisense-mediated angiotensinogen inhibition slows polycystic kidney disease in mice with a targeted mutation in Pkd2. Am J Physiol Renal Physiol. (2015) 308:F349–57. 10.1152/ajprenal.00478.2014 25537744 PMC4329486

[124] Yheskel M Lakhia R Cobo-Stark P Flaten A Patel V. Anti-microRNA screen uncovers miR-17 family within miR-17~92 cluster as the primary driver of kidney cyst growth. Sci Rep. (2019) 9:1920. 10.1038/s41598-019-38566-y 30760828 PMC6374450

[125] Lee EC Valencia T Allerson C Schairer A Flaten A Yheskel M. Discovery and preclinical evaluation of anti-miR-17 oligonucleotide RGLS4326 for the treatment of polycystic kidney disease. Nat Commun. (2019) 10:4148. 10.1038/s41467-019-11918-y 31515477 PMC6742637

[126] Mi Z Song Y Cao X Lu Y Liu Z Zhu X. Super-enhancer-driven metabolic reprogramming promotes cystogenesis in autosomal dominant polycystic kidney disease. Nat Metab. (2020) 2:717–31. 10.1038/s42255-020-0227-4 32694829

[127] Chiaravalli M Rowe I Mannella V Quilici G Canu T Bianchi V. 2-Deoxy-d-Glucose Ameliorates PKD Progression. J Am Soc Nephrol. (2016) 27:1958–69. 10.1681/ASN.2015030231 26534924 PMC4926967

[128] Magistroni R Boletta A. Defective glycolysis and the use of 2-deoxy-D-glucose in polycystic kidney disease: from animal models to humans. J Nephrol. (2017) 30:511–9. 10.1007/s40620-017-0395-9 28390001

[129] Ingram DK Roth GS. Glycolytic inhibition as a strategy for developing calorie restriction mimetics. Exp Gerontol. (2011) 46:148–54. 10.1016/j.exger.2010.12.001 21167272

[130] Carney EF. Ketosis slows the progression of PKD. Nat Rev Nephrol. (2020) 16:1. 10.1038/s41581-019-0226-4 31654043

[131] Warner G Hein KZ Nin V Edwards M Chini CC Hopp K. Food restriction ameliorates the development of polycystic kidney disease. J Am Soc Nephrol. (2016) 27:1437–47. 10.1681/ASN.2015020132 26538633 PMC4849816

[132] Kipp KR Rezaei M Lin L Dewey EC Weimbs T. A mild reduction of food intake slows disease progression in an orthologous mouse model of polycystic kidney disease. Am J Physiol Renal Physiol. (2016) 310:F726–31. 10.1152/ajprenal.00551.2015 26764208 PMC4835927

[133] Ramalingam H Kashyap S Cobo-Stark P Flaten A Chang CM Hajarnis S. A methionine-Mettl3-N(6)-methyladenosine axis promotes polycystic kidney disease. Cell Metab. (2021) 33:1234–47.e7. 10.1016/j.cmet.2021.03.024 33852874 PMC8172529

[134] Torres VE. Pro: tolvaptan delays the progression of autosomal dominant polycystic kidney disease. Nephrol Dial Transplant. (2019) 34:30–4. 10.1093/ndt/gfy297 30312438 PMC6657439

[135] Flanagan JU Yosaatmadja Y Teague RM Chai MZ Turnbull AP Squire CJ. Crystal structures of three classes of non-steroidal anti-inflammatory drugs in complex with aldo-keto reductase 1C3. PLoS ONE. (2012) 7:e43965. 10.1371/journal.pone.0043965 22937138 PMC3429426

[136] Aziz N Maxwell MM Brenner BM. Coordinate regulation of 11 beta-HSD and Ke 6 genes in cpk mouse: implications for steroid metabolic defect in PKD. Am J Physiol. (1994) 267:F791–7. 10.1152/ajprenal.1994.267.5.F791 7977782

[137] Lakhia R Yheskel M Flaten A Quittner-Strom EB Holland WL Patel V. PPARalpha agonist fenofibrate enhances fatty acid beta-oxidation and attenuates polycystic kidney and liver disease in mice. Am J Physiol Renal Physiol. (2018) 314:F122–31. 10.1152/ajprenal.00352.2017 28903946 PMC5866355

[138] Srivastava AK Jaganathan S Stephen L Hollingshead MG Layhee A Damour E. Effect of a smac mimetic (TL32711, Birinapant) on the apoptotic program and apoptosis biomarkers examined with validated multiplex immunoassays fit for clinical use. Clin Cancer Res. (2016) 22:1000–10. 10.1158/1078-0432.CCR-14-3156 26446940 PMC4755826

[139] Fan LX Zhou X Sweeney WE Jr Wallace DP Avner ED Grantham JJ. Smac-mimetic-induced epithelial cell death reduces the growth of renal cysts. J Am Soc Nephrol. (2013) 24:2010–22. 10.1681/ASN.2013020176 23990677 PMC3839552

[140] Bowman BT Rosner MH. Lixivaptan - an evidence-based review of its clinical potential in the treatment of hyponatremia. Core Evid. (2013) 8:47–56. 10.2147/CE.S36744 23874242 PMC3712664

[141] Woodhead JL Pellegrini L Shoda LK M Howell BA. Comparison of the hepatotoxic potential of two treatments for autosomal-dominant polycystic kidney diseaseusing quantitative systems toxicology modeling. Pharm Res. (2020) 37:24. 10.1007/s11095-019-2726-0 31909447 PMC6944674

[142] Fedeles SV Tian X Gallagher AR Mitobe M Nishio S Lee SH. A genetic interaction network of five genes for human polycystic kidney and liver diseases defines polycystin-1 as the central determinant of cyst formation. Nat Genet. (2011) 43:639–47. 10.1038/ng.860 21685914 PMC3547075

[143] Tan M Wettersten HI Chu K Huso DL Watnick T Friedlander S. Novel inhibitors of nuclear transport cause cell cycle arrest and decrease cyst growth in ADPKD associated with decreased CDK4 levels. Am J Physiol Renal Physiol. (2014) 307:F1179–86. 10.1152/ajprenal.00406.2014 25234309 PMC4254973

[144] Namli S Oflaz H Turgut F Alisir S Tufan F Ucar A. Improvement of endothelial dysfunction with simvastatin in patients with autosomal dominant polycystic kidney disease. Ren Fail. (2007) 29:55–9. 10.1080/08860220601038892 17365910

[145] Olson RJ Hopp K Wells H Smith JM Furtado J Constans MM. Synergistic genetic interactions between Pkhd1 and Pkd1 result in an ARPKD-like phenotype in murine models. J Am Soc Nephrol. (2019) 30:2113–27. 10.1681/ASN.2019020150 31427367 PMC6830782

[146] Koupepidou P Felekkis KN Kranzlin B Sticht C Gretz N Deltas C. Cyst formation in the PKD2 (1-703) transgenic rat precedes deregulation of proliferation-related pathways. BMC Nephrol. (2010) 11:23. 10.1186/1471-2369-11-23 20813037 PMC2936873

[147] Felekkis KN Koupepidou P Kastanos E Witzgall R Bai CX Tsiokas L. Mutant polycystin-2 induces proliferation in primary rat tubular epithelial cells in a STAT-1/p21-independent fashion accompanied instead by alterations in expression of p57KIP2 and Cdk2. BMC Nephrol. (2008) 9:10. 10.1186/1471-2369-9-10 18721488 PMC2533650

[148] Pandey P Brors B Srivastava PK Bott A Boehn SN Groene HJ. Microarray-based approach identifies microRNAs and their target functional patterns in polycystic kidney disease. BMC Genomics. (2008) 9:624. 10.1186/1471-2164-9-624 19102782 PMC2640396

[149] Chen WC Tzeng YS Li H. Gene expression in early and progression phases of autosomal dominant polycystic kidney disease. BMC Res Notes. (2008) 1:131. 10.1186/1756-0500-1-131 19099603 PMC2632667

[150] Allen E Piontek KB Garrett-Mayer E Garcia-Gonzalez M Gorelick KL Germino GG. Loss of polycystin-1 or polycystin-2 results in dysregulated apolipoprotein expression in murine tissues via alterations in nuclear hormone receptors. Hum Mol Genet. (2006) 15:11–21. 10.1093/hmg/ddi421 16301212 PMC1525254

[151] Kugita M Nishii K Morita M Yoshihara D Kowa-Sugiyama H Yamada K. Global gene expression profiling in early-stage polycystic kidney disease in the Han:SPRD Cy rat identifies a role for RXR signaling. Am J Physiol Renal Physiol. (2011) 300:F177–88. 10.1152/ajprenal.00470.2010 20926632

[152] Ameku T Taura D Sone M Numata T Nakamura M Shiota F. Identification of MMP1 as a novel risk factor for intracranial aneurysms in ADPKD using iPSC models. Sci Rep. (2016) 6:30013. 10.1038/srep30013 27418197 PMC4945931

[153] Cruz NM Song X Czerniecki SM Gulieva RE Churchill AJ Kim YK. Organoid cystogenesis reveals a critical role of microenvironment in human polycystic kidney disease. Nat Mater. (2017) 16:1112–9. 10.1038/nmat4994 28967916 PMC5936694

[154] Sun Y Liu Z Cao X Lu Y He C Liu J. Activation of P-TEFb by cAMP-PKA signaling in autosomal dominant polycystic kidney disease. Sci Adv. (2019) 5:eaaw3593. 10.1126/sciadv.aaw3593 31183407 PMC6551191

[155] Hogan MC Bakeberg JL Gainullin VG Irazabal MV Harmon AJ Lieske JC McCormick DJ. Identification of biomarkers for PKD1 using urinary exosomes. J Am Soc Nephrol. (2015) 26:1661–70. 10.1681/ASN.2014040354 25475747 PMC4483583

[156] Streets AJ Magayr TA Huang L Vergoz L Rossetti S Simms RJ. Parallel microarray profiling identifies ErbB4 as a determinant of cyst growth in ADPKD and a prognostic biomarker for disease progression. Am J Physiol Renal Physiol. (2017) 312:F577–88. 10.1152/ajprenal.00607.2016 28077374 PMC5504395

[157] Kunnen SJ Malas TB Semeins CM Bakker AD Peters DJM. Comprehensive transcriptome analysis of fluid shear stress altered gene expression in renal epithelial cells. J Cell Physiol. (2018) 233:3615–28. 10.1002/jcp.26222 29044509 PMC5765508

[158] Barrett T Wilhite SE Ledoux P Evangelista C Kim IF Tomashevsky M. NCBI GEO: archive for functional genomics data sets–update. Nucleic Acids Res. (2013) 41:D991–5. 10.1093/nar/gks1193 23193258 PMC3531084

[159] Haug K Cochrane K Nainala VC Williams M Chang J Jayaseelan KV. MetaboLights: a resource evolving in response to the needs of its scientific community. Nucleic Acids Res. (2020) 48:D440–4. 10.1093/nar/gkz1019 31691833 PMC7145518

[160] Brazma A Kapushesky M Parkinson H Sarkans U Shojatalab M. Data storage and analysis in ArrayExpress. Methods Enzymol. (2006) 411:370–86. 10.1016/S0076-6879(06)11020-4 16939801

[161] Perez-Riverol Y Csordas A Bai J Bernal-Llinares M Hewapathirana S Kundu DJ. The PRIDE database and related tools and resources in 2019: improving support for quantification data. Nucleic Acids Res. (2019) 47:D442–50. 10.1093/nar/gky1106 30395289 PMC6323896

[162] Shaar-Moshe L Hubner S Peleg Z. Identification of conserved drought-adaptive genes using a cross-species meta-analysis approach. BMC Plant Biol. (2015) 15:111. 10.1186/s12870-015-0493-6 25935420 PMC4417316

[163] Sweeney TE Haynes WA Vallania F Ioannidis JP Khatri P. Methods to increase reproducibility in differential gene expression via meta-analysis. Nucleic Acids Res. (2017) 45:e1. 10.1093/nar/gkw797 27634930 PMC5224496

[164] Chatterjee S Verma SP Pandey P. Profiling conserved biological pathways in Autosomal Dominant Polycystic Kidney Disorder (ADPKD) to elucidate key transcriptomic alterations regulating cystogenesis: A cross-species meta-analysis approach. Gene. (2017) 627:434–50. 10.1016/j.gene.2017.06.059 28676447

[165] co-chairs EAF Harris T Sandford R EAF members participants Roundtable. European ADPKD Forum multidisciplinary position statement on autosomal dominant polycystic kidney disease care: European ADPKD Forum and Multispecialist Roundtable participants. Nephrol Dial Transplant. (2018) 33:563–73. 10.1093/ndt/gfx327 29309655 PMC6018982

[166] De Rechter S Bockenhauer D Guay-Woodford LM Liu I Mallett AJ Soliman NA. ADPedKD: a global online platform on the management of children with ADPKD. Kidney Int Rep. (2019) 4:1271–84. 10.1016/j.ekir.2019.05.015 31517146 PMC6732756

[167] Schaefer F Mekahli D Emma F Gilbert RD Bockenhauer D Cadnapaphornchai MA. Tolvaptan use in children and adolescents with autosomal dominant polycystic kidney disease: rationale and design of a two-part, randomized, double-blind, placebo-controlled trial. Eur J Pediatr. (2019) 178:1013–21. 10.1007/s00431-019-03384-x 31053954 PMC6565642

[168] Carriazo S Perez-Gomez MV Cordido A Garcia-Gonzalez MA Sanz AB Ortiz A. Dietary care for ADPKD patients: current status and future directions. Nutrients. (2019) 11:1576. 10.3390/nu11071576 31336917 PMC6683072

